# A Comparative Analysis of Genetic Diversity and Structure in Jaguars (*Panthera onca*), Pumas (*Puma concolor*), and Ocelots (*Leopardus pardalis*) in Fragmented Landscapes of a Critical Mesoamerican Linkage Zone

**DOI:** 10.1371/journal.pone.0151043

**Published:** 2016-03-14

**Authors:** Claudia Wultsch, Lisette P. Waits, Marcella J. Kelly

**Affiliations:** 1 Department of Fish and Wildlife Conservation, Virginia Tech, Blacksburg, Virginia, United States of America; 2 Department of Fish and Wildlife Sciences, University of Idaho, Moscow, Idaho, United States of America; Smithsonian Conservation Biology Institute, UNITED STATES

## Abstract

With increasing anthropogenic impact and landscape change, terrestrial carnivore populations are becoming more fragmented. Thus, it is crucial to genetically monitor wild carnivores and quantify changes in genetic diversity and gene flow in response to these threats. This study combined the use of scat detector dogs and molecular scatology to conduct the first genetic study on wild populations of multiple Neotropical felids coexisting across a fragmented landscape in Belize, Central America. We analyzed data from 14 polymorphic microsatellite loci in 1053 scat samples collected from wild jaguars (*Panthera onca*), pumas (*Puma concolor*), and ocelots (*Leopardus pardalis*). We assessed levels of genetic diversity, defined potential genetic clusters, and examined gene flow for the three target species on a countrywide scale using a combination of individual- and population-based analyses. Wild felids in Belize showed moderate levels of genetic variation, with jaguars having the lowest diversity estimates (*H*_*E*_ = 0.57 ± 0.02; *A*_*R*_ = 3.36 ± 0.09), followed by pumas (*H*_*E*_ = 0.57 ± 0.08; *A*_*R*_ = 4.20 ± 0.16), and ocelots (*H*_*E*_ = 0.63 ± 0.03; *A*_*R*_ = 4.16 ± 0.08). We observed low to moderate levels of genetic differentiation for all three target species, with jaguars showing the lowest degree of genetic subdivision across the country, followed by ocelots and pumas. Although levels of genetic diversity and gene flow were still fairly high, we detected evidence of fine-scale genetic subdivision, indicating that levels of genetic connectivity for wild felids in Belize are likely to decrease if habitat loss and fragmentation continue at the current rate. Our study demonstrates the value of understanding fine-scale patterns of gene flow in multiple co-occurring felid species of conservation concern, which is vital for wildlife movement corridor planning and prioritizing future conservation and management efforts within human-impacted landscapes.

## Introduction

Carnivores are particularly vulnerable to loss and fragmentation of natural habitats because of their space and prey requirements, occurrence at low densities, and dependence on forest and/or habitats that provide sufficient cover [1, 2]. Landscape changes due to forest loss, degradation, and anthropogenic development can severely impact animal movement and the degree of genetic connectivity, consequently decreasing reproductive fitness and adaptive potential (e.g., [3, 4, 5]). Assessing genetic diversity and connectivity is important for understanding responses of wild populations to fragmented landscapes and anthropogenic disturbance. Sensitivity to these threats is species-specific, depending on dispersal characteristics, habitat specialization, trophic level, and other ecological traits (e.g., [2, 6]). Thus, comparative analysis of genetic connectivity among multiple species in the same landscape is particularly valuable as it may reveal common factors driving gene flow.

Neotropical carnivore guilds, which are among the most threatened worldwide [7] are understudied. The jaguar (*Panthera onca*), the largest Neotropical felid, which co-occurs with several other felids [e.g., puma (*Puma concolor*), ocelot (*Leopardus pardalis*)], has been extirpated from more than half of its historic range during the last 100 years, and its distribution continues to contract, mainly due to severe deforestation and habitat fragmentation, direct persecution, and loss of main prey species (e.g., [1, 8–10]). Mesoamerican jaguar populations are reduced to one third of their historic range, and 75% of the remaining jaguar populations have declined in numbers and are potentially highly fragmented [10, 11]. To prevent further loss of these predators, it is crucial to conduct efficient monitoring of their wild populations, and gather valuable scientific data to inform conservation and management of this internationally protected species (listed as ‘Near Threatened’ under the International Union for Conservation of Nature [IUCN], as an *Appendix I* species under the Convention on International Trade of Endangered Species of Wild Fauna and Flora [CITES]). Co-occurring pumas and ocelots face similar challenges to their survival. Pumas have also disappeared from large portions of their historic range and are listed as a ‘Least Concern’ species under IUCN and as an *Appendix I* (eastern and Central American subspecies) and an *Appendix II* (remaining subspecies) species under CITES [1, 12]. Ocelots, the only medium-sized felid found in the Neotropics, are listed as a ‘Least Concern’ species under IUCN and are included as an *Appendix I* species under CITES [1, 8, 13]. Wild populations of all three Neotropical felid species are considered understudied across Mesoamerica, a region that faces one of the highest deforestation rates worldwide [14, 15].

Molecular genetic approaches provide powerful tools to assess the conservation status of multiple species of concern by monitoring genetic diversity and connectivity. Advancements in noninvasive genetic monitoring techniques, including the use of molecular scatology and scat detector dogs [16–21], have made it more powerful and feasible to genetically study multiple elusive forest carnivores simultaneously in tropical environments, without having to physically capture and handle animals. The use of professionally trained scat detector dogs significantly increased scat-collection rates for several recent molecular scatology studies conducted in tropical environments (e.g., [17, 20, 21]). Implementing conservation genetic studies for multiple species simultaneously is more efficient and enables better identification of landscapes of high conservation concern to all species, thus aiding in wildlife movement corridor planning and conservation management efforts (e.g., [5, 22]). Nonetheless, molecular population genetics studies of jaguars and other Neotropical felids are still relatively rare (e.g., [23, 24–28]). Only a handful of current noninvasive genetic studies sample wild populations of multiple felid species and most of them are based in South America (e.g., [20, 21, 28, 29, 30]).

To address this knowledge gap, we conducted a 4-year (2007–2010) noninvasive genetic study on jaguars and two co-occurring felids, pumas and ocelots, using fecal DNA samples collected with the aid of a scat detector dog across several study sites in Belize, Central America. Belize and its neighboring countries, Guatemala, and Mexico, are part of La Selva Maya (the Maya Forest), the largest remaining tropical forest in Mesoamerica, and the largest intact forest north of the Amazon, representing a critical link in the Mesoamerican Biological Corridor. Like other Mesoamerican countries, Belize has been experiencing widespread land conversion due to agricultural and urban development, logging, cattle-ranching, and natural disasters (e.g., hurricanes, forest fires), that have increased deforestation rates (2.3% yearly) above the Central American average (1.2% yearly) over the last two decades [31, 32]. We hypothesized that genetic diversity and connectivity would be reduced for felids in the most northern and southern protected areas, which are more isolated and face one of the highest deforestation rates within the country [31]. We also expected to see lowered levels of genetic connectivity between protected areas separated by human-constructed barriers (e.g., intensive agriculture, roads, and urban development) such as in central and northern Belize. Furthermore, we hypothesized that jaguars would exhibit higher levels of genetic differentiation than pumas, since pumas, especially males reportedly are long-distance dispersers and are more likely to move through disturbed and fragmented areas than jaguars (e.g., [33, 34]). To test these hypotheses and assess the conservation status of wild felids in Belize, we specifically aimed to: (a) estimate levels of genetic diversity within different regions of the country, (b) examine patterns and spatial scale of genetic structure using multiple individual- and population-based analyses, and (c) assess contemporary gene flow and dispersal movements for all three target species. We discuss the implications of our results to support current and future conservation and management efforts, including wildlife movement corridors for wild felids in Belize.

## Materials and Methods

### Ethics Statement

Permission to undertake fieldwork and sample collection was obtained from the Belize Forest Department. No review from the ethics committee was required, as our research work applied noninvasive genetic sampling methods and did not involve any direct manipulation or disturbance of animals.

### Study Area

**Fig 1:**
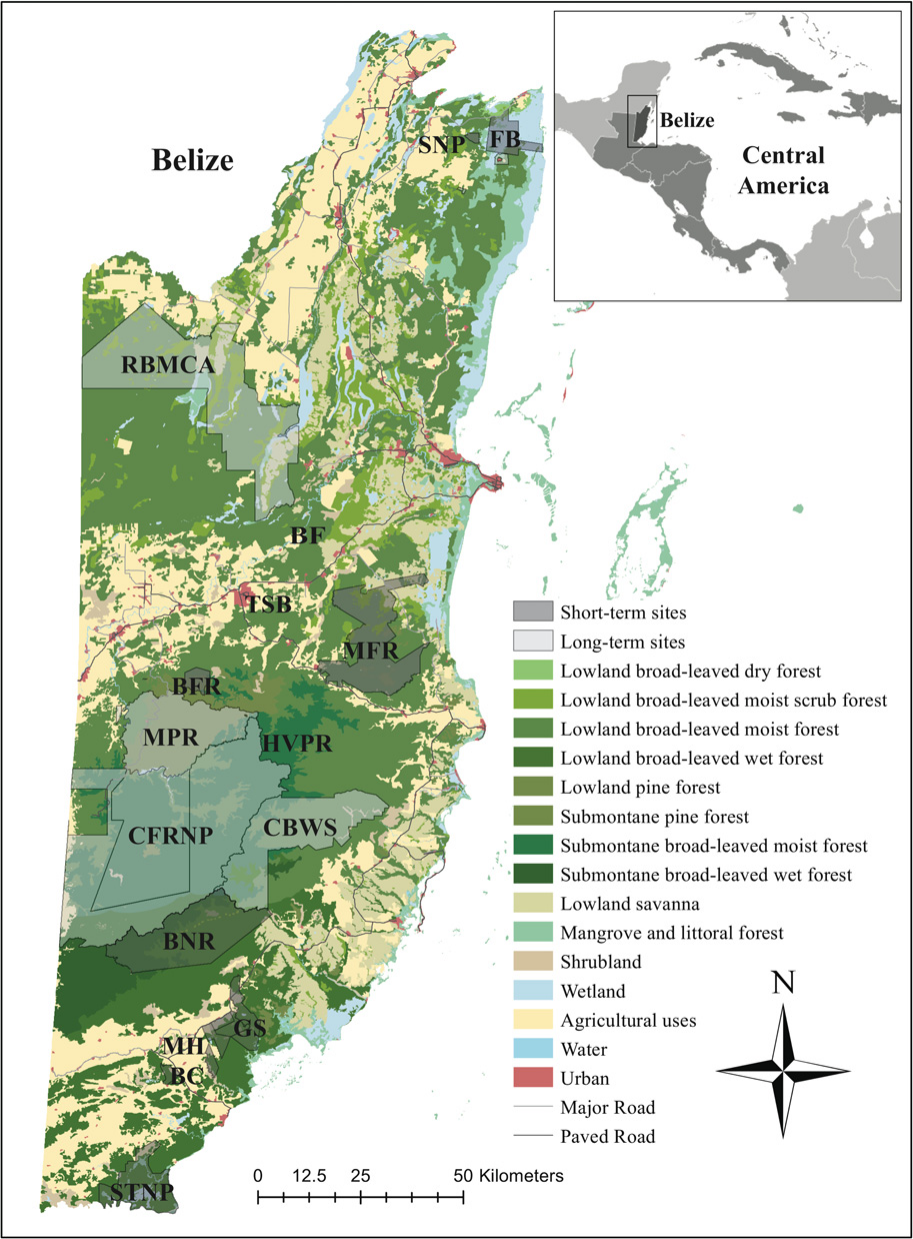
Study area map of short- and long-term survey sites for Neotropical felids across various geographical regions in Belize. Geographical regions covered include (1) the north (FB, Fireburn/Balam Na Nature Reserve; SNP, Shipstern Nature Reserve), (2) north-central (RBCMA, Rio Bravo Conservation and Management Area; BF, Big Falls; MFR, Manatee Forest Reserve; TSB, Tiger Sandy Bay), (3) central (MPR, Mountain Pine Ridge Forest Reserve; CFRNP, Chiquibul Forest Reserve and National Park; BFR, Bull Run Farm; HVPR, Hidden Valley Private Reserve), (4) south-central (CBWS, Cockscomb Basin Wildlife Sanctuary), and (5) south (BNR, Bladen Nature Reserve; BC, Boden Creek Ecological Preserve; GS, Golden Stream Corridor Preserve; MH, Machaca Hills; STNP, Sarstoon-Temash National Park).

We conducted 2–3 month long scat surveys across 5 study sites (Mountain Pine Ridge Forest Reserve–MPR, Rio Bravo Conservation and Management Area–RBCMA, Cockscomb Basin Wildlife Sanctuary–CBWS, Chiquibul Forest Reserve and National Park–CFRNP, Fireburn/Balam Na Nature Reserve–FB) and 2- to 10-day surveys at several other sites (Big Falls–BF, Bladen Nature Reserve—BNR, Boden Creek Ecological Preserve—BC, Bull Run Farm–BRF, Golden Stream Corridor Preserve—GS, Hidden Valley Private Reserve–HVPR, Machaca Hills–MH, Manatee Forest Reserve–MFR, Sarstoon-Temash National Park—STNP, Shipstern Nature Preserve–SNP, Tiger Sandy Bay—TSB) from 2007–2010 across Belize, Central America (17°15’ N, 88°45’ W; Fig 1). All sites except BF, BRF, HVPR, MH and TSB are part of the national system of protected areas in Belize. For the genetic diversity and indirect genetic structure study, we predefined groups of individuals based on geographical regions and potential barriers to gene flow, including natural (e.g., mountain ranges) and anthropogenic landscape (e.g., urban and agricultural areas, roads) features. For jaguars and pumas, we evaluated five geographical regions in Belize, which included the following study sites: north (FB, SNP), north-central (RBCMA, BF, MFR, TSB), central (MPR, CFRNP, BFR, HVPR), south-central (CBWS), and south (BNR, BC, GS, MH, STNP) (Fig 1). For ocelots, due to low sample size, we evaluated felids detected in the north (FB, SNP, RBCMA, BF, MFR, TSB), and south (MPR, CFRNP, BFR, HVPR, BNR, BC, GS, MH, STNP) (Fig 1). Across study sites, elevation ranges from 0 to 1120 m, and mean annual rainfall varies from 1524 mm in the north to 4064 mm in the south with a pronounced wet season from June to December. Average annual temperatures fluctuate between 17.7 and 31.3°C. A high diversity of native habitat types occurs within the study sites, including lowland and submontane broadleaf moist and wet forests (both primary and secondary growth), lowland and submontane pine forests, mangrove and littoral forests, lowland savannah, shrub land, and wetland swamps called bajos.

### Fecal DNA sampling and genotyping

Fecal samples were detected opportunistically in the field by using a professionally trained scat detector dog (PackLeader LLC, Gig Harbor, WA, US) following the study design and sampling protocols described by Wultsch, Waits [35]. DNA extraction was carried out for all fecal samples detected by the scat detector dog following protocols in Wultsch, Waits [20]. Fecal samples were assigned to feline species using species-specific microsatellite alleles identified in 14 polymorphic microsatellite loci, confirmed by mitochondrial DNA sequencing [20]. The microsatellite genotypes were also used to identify individuals, and by combining and arranging these loci with two additional DNA markers associated with the Y sex chromosome carried by males (Zn, Zn-finger; Amel, Amelogenin), but not by females [36], into three multiplexes (S1 Materials and Methods), we were also able to determine the sex of individuals. All extractions and polymerase chain reactions (PCR) included negative controls. To finalize consensus genotypes, we conducted an average of 4.29 (SD ± 1.84) PCR replicates per locus and sample. In addition, a multi-tube approach was used, where at least three identical PCR results were required for homozygote genotypes, and each allele had to be observed at least twice to confirm heterozygote genotypes. The genetic analysis was conducted at a laboratory specialized in the analysis of noninvasive genetic samples (Laboratory for Ecological, Evolutionary, and Conservation Genetics, University of Idaho, Moscow, ID, US).

### Genetic diversity

We used GenAlEx, version 6.41 [37] to assess genetic variation per species and geographical region (north, north-central, central, south-central, and south for jaguars and pumas; north and south for ocelots) across all loci by estimating the number of alleles (*N*_*A*_), the number of private alleles (*A*_*P*_), observed (*H*_*O*_) and expected heterozygosities (*H*_*E*_), and inbreeding coefficients (*F*_*IS*_). Additionally, we determined allelic richness (*A*_*R*_) using the rarefaction method with HP-RARE, version 1.0 [38]. Statistical differences between groups were evaluated using non-parametric Kruskal-Wallis rank-sum tests in R, version 3.1.3 [39]. We tested for linkage disequilibrium and departures from Hardy-Weinberg proportions using exact tests in GENEPOP, version 4.1 [40] with default settings for Markov chain parameters. Results for multiple significance tests were adjusted by applying a sequential Bonferroni approach [41].

### Fine-scale genetic structure and contemporary gene flow

To determine whether felid species in Belize represent single panmictic populations or several subdivided populations, we used multiple approaches to assess genetic structure and levels of gene flow indirectly. We used *F*-statistics to examine the degree of genetic differentiation by calculating pairwise *F*_*ST*_ values [42] between groups of felids detected across the five geographical regions. To account for high mutation rates of microsatellites, which may underestimate genetic differentiation using *F*_*ST*_ values [43], we also calculated pairwise standardized *G’*_*ST*_ estimates [44] using GenAlEx, version 6.41 [37]. Genetic differentiation was further examined via analysis of molecular variation (AMOVA, [45]) in Arlequin, version 3.5.1.3 [46]. Different hypotheses about hierarchical genetic differentiation for each species were tested, including genetic subdivision by sampling geographical (scenario A: North–FB, SNP; North-Central–BF, RBCMA, MFR, TSB; Central–BFR, CFRNP, HVPR, MPR; South-Central–CBWS; South–BC, BNR, GS, MH, STNP) and north and south regions (scenario B: North–FB, SNP, BF, RBCMA, MFR, TSB; South–BFR, CFRNP, HVPR, MPR, CBWS, BC, BNR, GS, MH, STNP) across Belize. To complement these analyses, we also conducted exploratory multivariate analyses, including discriminant analysis of principal components (DAPC), using *adegenet*, version 1.4.2 [47] in R, version 3.1.3 [39].

To assess gene flow more directly and gain insight into contemporary rates of genetic change, we conducted several different types of individual-based assignment tests using aspatial [48, 49] and spatial Bayesian clustering techniques [50–52]. We applied aspatial Bayesian clustering in STRUCTURE, version 2.3.4 [48, 49] to determine the optimal number of genetic groups/clusters (*K*) by running 2,000,000 Markov chain Monte Carlo (MCMC) iterations after a burn-in of 200,000 replicates with 10 independent runs per *K* (ranging from 1 to 10 for each species), using the admixture model with correlated allele frequencies, which estimates fractions of individual genomes that belong to different ancestry groups. We also applied the same model adding sampling locations as prior (LOCPRIOR, [49]). The optimal *K* value was chosen by calculating the mean posterior probability for each *K* value, LnP(D), which is based on estimated maximum log-likelihood values [48]. We also calculated Δ*K* values (the rate of change in the log probability of data between successive *K* values) as suggested by Evanno et al. [53] using STRUCTURE-HARVESTER, version 0.6.94 [54]. Individual membership assignments estimated in STRUCTURE were averaged using CLUMPP, version 1.2.2 [55] with a Greedy algorithm and 10,000 random permutations. Furthermore, we implemented spatially-explicit Bayesian clustering using GENELAND, version 4.0.3 [52] in R, version 3.1.3 [39]. We used the spatially explicit model (1,000,000 MCMC iterations, thinning = 100), and set the number of potential populations to *K* equals 1 to 10. We applied the uncorrelated frequency model, which uses the Dichirilet distribution [48] to model allele frequencies as recommended by Guillot et al. [52]. The uncertainty associated with spatial coordinates was set to 20 m. Next, we performed ten additional runs for the selected *K* value, using the same parameters. To verify the consistency of this analysis, we repeated the analysis three times total and compared the results with each other. After finding the optimal *K* value with STRUCTURE and GENELAND, individuals were assigned to distinct genetic clusters using the percentage of genotypes' ancestry (*Q*; scores ≥ 70% indicate assignment to a genetic cluster and scores < 70% admixture).

To characterize countrywide spatial genetic structure, we also examined isolation by distance (IBD) and spatial autocorrelation patterns. First, we determined whether a significant correlation existed between pairwise codominant genotypic and geographical distances by applying simple Mantel tests for each species and sex with 10,000 permutations using *ecodist*, version 1.2.9 [56] in R, version 3.1.3 [39]. Second, spatial autocorrelation analysis was conducted in GenAlEx, version 6.41 [37] to examine the spatial extent of genetic structure and to determine if dispersal patterns were sex-biased. We correlated pairwise geographical and genetic distance matrices for each sex and species and generated autocorrelation coefficients (*r*) similar to Moran’s I coefficient for each spatial distance class [57], which were visualized as correlograms. The distance classes ranging from 5 to 250 km were chosen based on the distribution of geographic distances among individuals. Spatial genetic structure is tested against the null hypothesis of no autocorrelation (*r* = 0) by generating 95% confidence intervals (CI) for each distance class via permutation (9,999 simulations) and bootstrapping (999 repeats). Within a given correlogram, significant spatial autocorrelation was confirmed only when a positive *r*-value fell outside the 95% CI (derived from the permutation test), and when the 95% CI about *r* (derived from bootstrapping) did not intercept the axis of *r* = 0 as described by Peakall, Ruibal [57]. The extent of non-random spatial genetic structure was based on the location of the first x-intercept (e.g., [57]).

Furthermore, to indirectly infer patterns of contemporary gene flow, we calculated maximum-likelihood estimates of pairwise relatedness coefficients (r) ranging from 0 (unrelated) to 0.5 (parent-offspring, full-siblings) among individual felids for all three target species within and among different sites in ML-RELATE [58]. Statistical differences between groups were evaluated using non-parametric Wilcoxon and Kruskal-Wallis rank-sum tests in R, version 3.1.3 [39].

### Detection of migrants and contemporary migration rates

To assess levels of contemporary migration (i.e. within the last few generations) per species and among the five different geographical regions within Belize, we used two different approaches. First, we assessed contemporary dispersal by determining first-generation migrants in GENECLASS, version 2.0, which applies MCMC resampling algorithms to compute individual probabilities of being a resident (i.e., not a first-generation migrant) to each reference population [59, 60]. The analysis is based on maximum likelihood estimation (*Lhome*:*Lmax*, the ratio of *L_home* to the highest likelihood value among all available population samples including the population where the individual was sampled [*L_max*]) [60]. We applied the Bayesian criterion of Rannala and Mountain [61] in combination with the MCMC resampling method of Paetkau, Slade [60]. We simulated 10,000 individuals and selected an alpha level of 0.01. Second, we estimated contemporary directional migration rates (i.e. migration events that occurred within the last few generations) using Bayesian inference framework implemented in BayesAss+, version 3.0 [62]. We conducted the analysis using 3,000,000 iterations, 1,000,000 iterations burn-in, and a sampling frequency of 2000. We used the default delta value of 0.15 for allele frequencies, migration rate, and inbreeding. We conducted ten independent runs of the analysis to confirm the consistency of migration rate estimates. Bidirectional migration rates were visualized as circos plots using *circlize*, version 0.3.4 [63] in R, version 3.1.3 [39].

## Results

### Microsatellite genotyping

Of 1053 scat samples collected, 530 (50%) were successfully identified to species and individual. At the individual level, we detected 65 jaguars (57 males, 8 females), 54 pumas (30 males, 24 females) and 30 ocelots (5 males, 25 females) (Table 1). In total, jaguars were genetically “captured” 307 times, pumas 161 times, and ocelots 62 times.

**Table 1:** Sampling summary.

	*Panthera onca*			*Puma concolor*			*Leopardus pardalis*		
Region	*n*	Males	Females	*n*	Males	Females	*n*	Males	Females
North	8	8	0	13	7	6	15	4	11
**North-Central**	16	13	3	19	8	11	---	---	---
**Central**	18	14	4	10	6	4	---	---	---
**South-Central**	15	15	0	7	6	1	---	---	---
**South**	8	7	1	5	3	2	15	1	14
Total	65	57	8	54	30	24	30	5	25

Number of individual (*n*) jaguars, pumas, and ocelots and number of males and females per species detected across five geographical regions (north, north-central, central, south-central, south) in Belize, Central America.

### Genetic diversity

Countrywide genetic diversity estimates were different among species (Kruskal-Wallis rank-sum tests, *A*_*R*_, *H* = 13.19, *P* = 0.002; *A*_*P*,_ *H* = 7.52, *P* = 0.023; *H*_*e*_, *H* = 4.55, *P* = 0.103), and highest for ocelots followed by pumas and jaguars (Table 2). Diversity estimates did not differ significantly across geographical regions for jaguars (Kruskal-Wallis rank-sum tests, *A*_*R*_, *H* = 0.75, *P* = 0.95; *A*_*P*,_ *H* = 4.54, *P* = 0.34; *H*_*e*_, *H* = 1.20, *P* = 0.88) or ocelots (Kruskal-Wallis rank-sum tests, *A*_*R*_, *H* = 0.03, *P* = 0.87; *A*_*P*,_ *H* = 0.35, *P* = 0.56; *H*_*e*_, *H* = 0.10, *P* = 0.75). Pumas had significantly lower allelic richness estimates at the northern site compared with most other regions (Kruskal-Wallis rank-sum test, *A*_*R*_, *H* = 11.10, *P* = 0.03), but did not differ for other diversity estimates (Kruskal-Wallis rank-sum test, *H*_*e*_, *H* = 8.10, *P* = 0.09; *A*_*P*,_ *H* = 4.58, *P* = 0.33). *F*_*IS*_ values for all target species and geographical regions were positive and at low to moderate levels, ranging from 0.00 to 0.08 for jaguars, 0.01 to 0.12 for pumas, and 0.04 to 0.14 for ocelots (Table 2). After sequential Bonferroni corrections, loci FCA043 (*P* < 0.000) and F98 (*P* = 0.011) for pumas, and loci FCA391 (*P* = 0.001), FCA275 (*P* < 0.000), and FCA741 (*P* = 0.011) for ocelots significantly deviated from Hardy-Weinberg expectations. Significant linkage disequilibrium after sequential Bonferroni correction (P ≤ 5.00E-04) was only detected among one pair of loci (FCA096 and FCA441) in jaguars.

**Table 2:** Summary statistics of genetic diversity for *Panthera onca*, *Puma concolor*, *and Leopardus pardalis* in Belize.

Region	*Panthera onca* (*n* = 65)							*Puma concolor* (*n* = 54)							*Leopardus pardalis* (*n* = 30)						
	*N*	*N*_*A*_	*A*_*P*_	*A*_*R*_	*H*_*O*_	*H*_*E*_	F_IS_	*N*	*N*_*A*_	*A*_*P*_	*A*_*R*_	*H*_*O*_	*H*_*E*_	F_IS_	*N*	*N*_*A*_	*A*_*P*_	*A*_*R*_	*H*_*O*_	*H*_*E*_	F_IS_
**North**	8	3.29	0	3.22	0.55	0.54	0.05	13	5.00	4	3.98	0.57	0.58	0.06	15	5.00	16	4.10	0.61	0.61	0.04
**N.-Central**	16	3.93	3	3.37	0.58	0.58	0.03	19	5.86	11	4.24	0.66	0.64	0.01	---	---	---	---	---	---	---
**Central**	18	4.14	4	3.42	0.56	0.58	0.06	10	4.79	7	4.23	0.61	0.63	0.09	---	---	---	---	---	---	---
**S.-Central**	15	4.00	4	3.32	0.58	0.55	0.00	7	4.21	6	4.14	0.61	0.55	0.01	---	---	---	---	---	---	---
**South**	8	3.64	2	3.46	0.59	0.59	0.08	5	2.43	4	4.43	0.58	0.44	0.12	15	5.21	19	4.21	0.61	0.66	0.14
**Mean**		3.80	2.60	3.36	0.57	0.57	0.04		4.46	6.40	4.20	0.60	0.57	0.06		5.11	17.50	4.16	0.61	0.63	0.09
**SD**		0.34	1.67	0.09	0.01	0.02	0.03		1.28	2.88	0.16	0.03	0.08	0.05		0.15	2.12	0.08	0.00	0.03	0.07

Diversity estimates were calculated by species and geographical region (north, north-central, central, south-central, south). *N*, number of individuals; *N*_*A*_, number of alleles; *A*_*P*_, number of private alleles; *A*_*R*_, allelic richness using the rarefaction method; *H*_*O*_, observed heterozygosity; *H*_*E*_, expected heterozygosity; F_IS_, inbreeding coefficient; *n*, number of individual felids.

### Fine-scale genetic structure and contemporary gene flow

**Fig 2:**
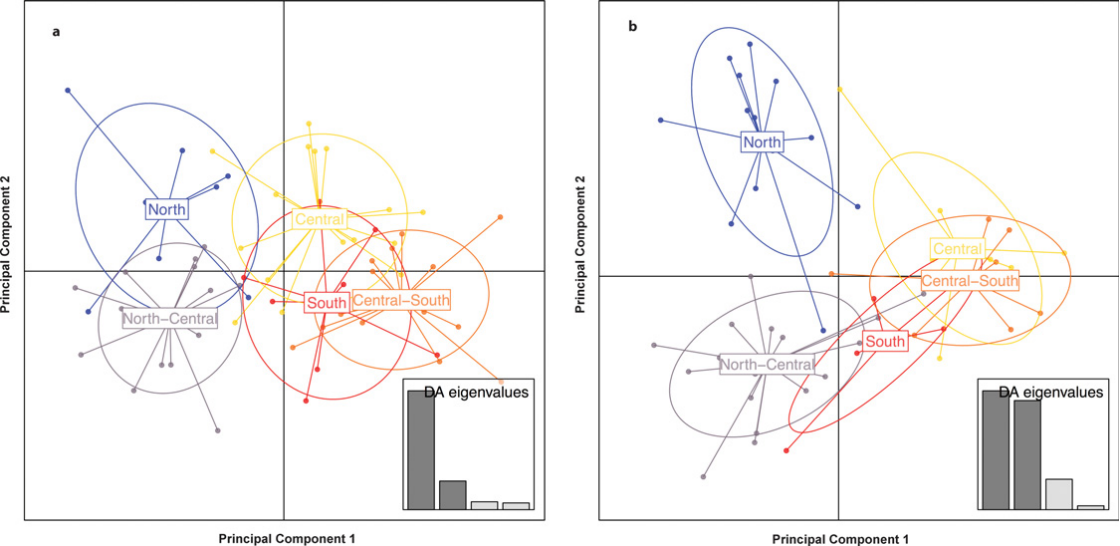
Discriminant analysis of principal components (DAPC) of Neotropical felids in Belize. Analysis was implemented with (a) *Panthera onca* and (b) *Puma concolor* genotypes detected across five geographical regions (north, north-central, central, south-central, south) within Belize using *adegenet*, version 1.4.2 [47] in software R, version 3.1.3 [39]. Scatterplots show the first two principal components. Points represent individual genotypes, and geographical groups of jaguars and pumas are represented through 95% inertia ellipses in different colors. The barplot (bottom-right) graphs eigenvalues of the first four principal components in relative magnitude.

Pairwise *F*_*ST*_ values between most geographical regions were significantly different from zero for all three species (Table 3). Our results suggested low to moderate genetic differentiation for all three target species, ranging from 0.029 to 0.063 for jaguars, and 0.038 to 0.131 for pumas. For ocelots, a *F*_*ST*_ value of 0.049 was detected between the northern and southern region. For jaguars, levels of genetic differentiation were moderate between north and south-central (*F*_*ST*_ = 0.063), and north-central and south-central (*F*_*ST*_ = 0.050) sites. For pumas, moderate genetic differentiation was detected among most regions. *F*_*ST*_ values were highest between north and south (*F*_*ST*_ = 0.131), followed by south-central and south (*F*_*ST*_ = 0.107), central and south (*F*_*ST*_ = 0.100), north and south-central (*F*_*ST*_ = 0.084), and north- and south-central (*F*_*ST*_ = 0.061) sites. Pairwise *G’*_*ST*_ estimates indicated moderate to high levels of genetic differentiation for all three target species across several study sites (Table 3). The AMOVA results for jaguars, pumas, and ocelots between genetic clusters defined by geographical regions within Belize are given in Table 4. All three comparisons indicated low but significant genetic differentiation. The AMOVA test showed highest levels of genetic variation within sampling sites and regions (> 85%), indicating that genetic differentiation among sampling sites and regions is low for all three target species. DAPC analysis suggested that jaguars within Belize form two groups with low levels of genetic differentiation as north and north-central jaguars were slightly separated from the remaining three regions (Fig 2A). For pumas, DAPC grouped individuals in three groups corresponding to the north, north-central, and central/central-south/south geographical regions (Fig 2B).

**Table 3:** Genetic differentiation of *Panthera onca*, *Puma concolor*, *and Leopardus pardalis* in Belize.

**(a) *Panthera onca*** (*n* = 65)	**North**	**North-Central**	**Central**	**South-Central**	**South**
North (*n* = 8)	---	0.031NS	0.033NS	0.063**	0.048NS
North-Central (*n* = 16)	0.021NS	---	0.029*	0.050**	0.032NS
Central (*n* = 18)	0.032NS	0.06*	---	0.018NS	0.019NS
South-Central (*n* = 15)	0.166**	0.153**	0.002NS	---	0.022NS
South (*n* = 8)	0.056NS	0.027NS	-0.037NS	-0.024NS	---
**(b) *Puma concolor*** (*n* = 54)	**North**	**North-Central**	**Central**	**South-Central**	**South**
North (*n* = 13)	---	0.041**	0.053**	0.061**	0.131**
North-Central (*n* = 19)	0.115**	---	0.038**	0.050*	0.084**
Central (*n* = 10)	0.145**	0.093**	---	0.046NS	0.100**
South-Central (*n* = 7)	0.135**	0.111*	0.051NS	---	0.107*
South (*n* = 5)	0.343**	0.179**	0.216***	0.192**	---
**(c) *Leopardus pardalis*** (*n* = 30)		**North**		**South**	
North (*n* = 15)		---		0.049**	
South (*n* = 15)		0.173**		---	

Pairwise *F*_*ST*_ and Hedrick’s *G’*_*ST*_ estimates and associated *P*–values (NS, not significant

*P < 0.05

**P < 0.005) for: (a) *Panthera onca*, (b) *Puma concolor*, and (c) *Leopardus pardalis* among five geographical regions (north, north-central, central, south-central, south) in Belize obtained in GenAlEx, version 6.41 [37]. Above the diagonal (pairwise *F*_*ST*_ estimates), below the diagonal (pairwise *G’*_*ST*_ estimates). *n*, number of individuals.

**Table 4:** Hierarchical analysis of molecular variation for *Panthera onca*, *Puma concolor*, *and Leopardus pardalis* in Belize.

	Source of variation	d.f.	Sum of squares	VC	% Variation	*F*_*ST*_	*P-value*
***Panthera onca***	Among sampling sites	5	11.62	-0.05	-3.29%	0.061	0.002
**A**	Among populations within sampling sites	1	2.75	0.15	9.43%		
	Within sampling sites	58	86.15	1.49	93.86%		
	Total	64	100.52	1.58	100%		
	Among regions	1	3.97	0.06	3.87%	0.079	0.002
**B**	Among populations within regions	5	10.40	0.06	4.02%		
	Within regions	58	86.15	1.49	92.11%		
	Total	64	100.52	1.61	100%		
***Puma concolor***	Among sampling sites	4	9.60	0.04	2.50%	0.080	<0.000
**A**	Among populations within sampling sites	1	1.70	0.08	5.51%		
	Within sampling sites	48	62.53	1.30	91.99%		
	Total	53	73.83	1.42	100%		
	Among regions	1	3.04	0.03	2.09%	0.087	0.001
**B**	Among populations within regions	4	8.26	0.09	6.58%		
	Within regions	48	62.53	1.30	91.33%		
	Total	53	73.83	1.43	100%		
***Leopardus pardalis***	Among sampling sites	2	7.71	0.11	5.54%	0.132	<0.000
**A**	Among populations within sampling sites	1	2.37	0.15	7.66%		
	Within sampling sites	26	43.23	1.66	86.81%		
	Total	29	53.30	1.92	100%		
**B**	Among regions	1	4.03	0.03	1.42%	0.130	<0.000
	Among populations within regions	2	6.04	0.22	11.58%		
	Within regions	26	43.23	1.66	86.99%		
** **	Total	29	53.30	1.91	100%		

AMOVA is based on *F*_*ST*_ estimates in 14 microsatellite loci testing two hypotheses for genetic divergence, including scenario A (subdivision by sampling site): North–FB, SNP; North-Central–BF, RBCMA, MFR, TSB; Central–BFR, CFRNP, HVPR, MPR; South-Central–CBWS; South–BC, BNR, GS, MH, STNP), and scenario B (subdivision by region): North–FB, SNP, BF, RBCMA, MFR, TSB; South–BFR, CFRNP, HVPR, MPR, CBWS, BC, BNR, GS, MH, STNP. d.f., degrees of freedom; VC, variance components; *F*_*ST*_, fixation index as measure of genetic differentiation.

**Fig 3:**
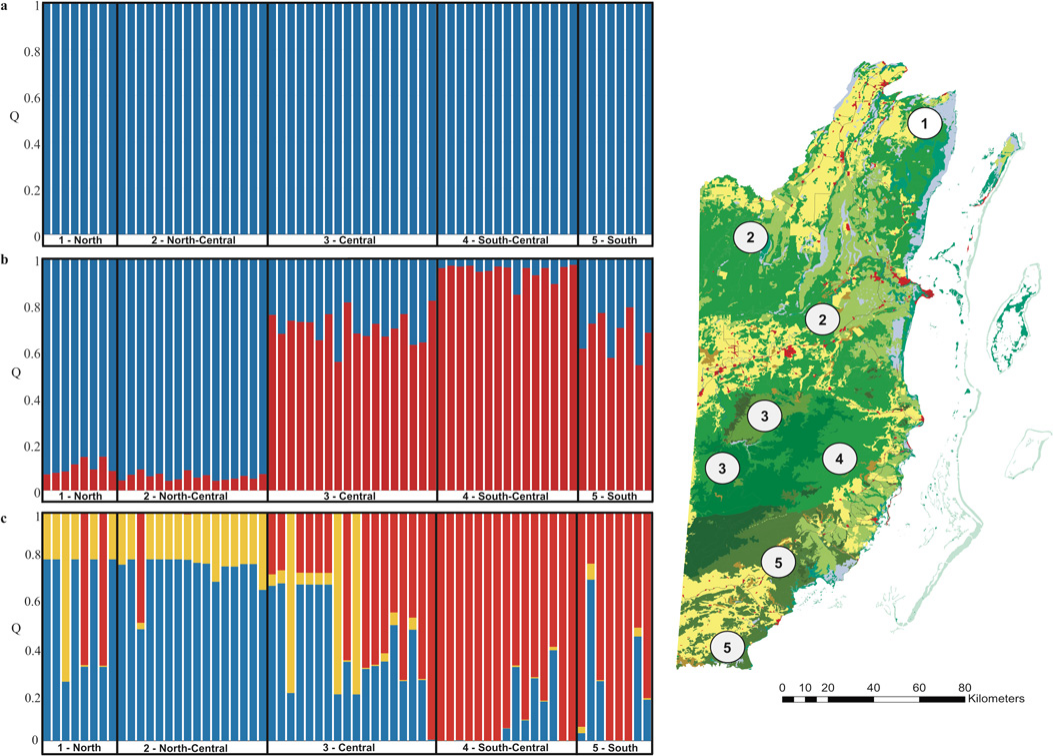
Genetic structure of *Panthera onca* in Belize. Inference for number of genetic clusters (*K*) was based on mean log likelihood LnP(D), using the admixture model with correlated allele frequencies without (a) and (b) with prior sampling location information obtained in STRUCTURE, version 2.3.3 [48], and (c) spatially-explicit Bayesian clustering assignment in GENELAND, version 4.0.3. [52]. Within the barplots each bar represents one individual felid and the color of the bar represents the % of membership (*Q*) the individual belongs to different genetic clusters.

Non-spatial Bayesian clustering analysis in STRUCTURE indicated no genetic structure for jaguars with *K* = 1 as the most probable number of genetic clusters using the admixture model with correlated allele frequencies (Fig 3A, S1A Fig). However, when prior location information of the origin of the scat samples was included (LOCPRIOR model), the number of genetically distinguishable groups detected by STRUCTURE was *K* = 2, roughly grouping sites into a northern and southern cluster consistent with DAPC analyses (Fig 3B, S1B Fig). Jaguars detected within the north/north-central regions had a high membership assignment to the northern genetic cluster (mean *Q* = 0.98, range 0.92–0.99). More than half of the jaguars (56%) detected in the central region were assigned to the southern genetic cluster (mean *Q* = 0.70, range 0.55–0.81), whereas the rest had partial membership in the northern and southern genetic cluster. South-central jaguars from the Cockscomb Basin Wildlife Sanctuary were fully assigned to their region (mean *Q* = 0.96, range 0.84–0.98). Jaguars detected in southern Belize were primarily (75%) of admixed ancestry (mean *Q* = 0.67, range 0.62–0.73). Finally, our analysis applying spatially-explicit Bayesian clustering in GENELAND resulted in *K* = 3 as the most probable number of genetic clusters but no new spatial groups were defined (Fig 3C). The boundaries of the inferred genetic clusters correspond to the main geographical regions within the country roughly separating jaguars into a northern (mean *Q* = 0.71) and southern group (mean *Q* = 0.87), with several jaguar individuals in central Belize (Mountain Pine Ridge Forest Reserve, Chiquibul Forest Reserve and National Park) forming a cline of admixed ancestry. Southern jaguars exhibited lower levels of genetic admixture in comparison to the north, which was particularly evident at Cockscomb Basin Wildlife Sanctuary, where most jaguars (~ 70%) had strong membership assignments (*Q* > 0.90), thus very low levels of admixed ancestry.

**Fig 4:**
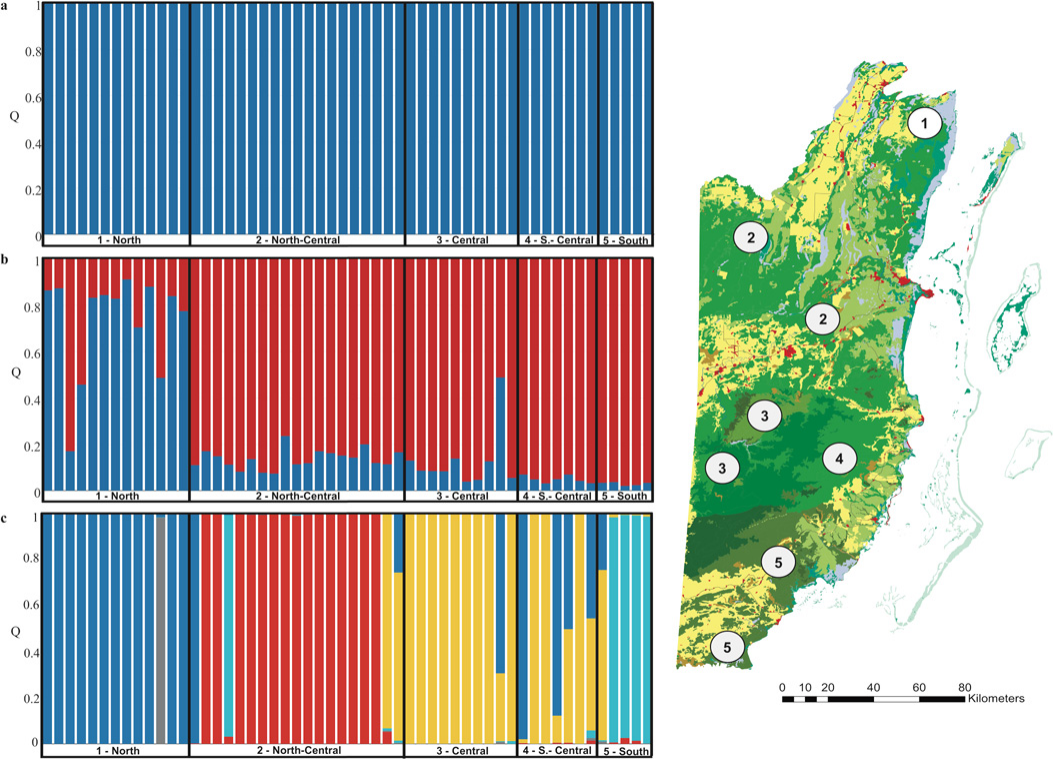
Genetic structure of *Puma concolor* in Belize. Inference for number of genetic clusters (*K*) was based on mean log likelihood LnP(D), using the admixture model with correlated allele frequencies without (a) and (b) with prior sampling location information obtained in STRUCTURE, version 2.3.3 [48], and (c) spatially-explicit Bayesian clustering assignment in GENELAND, version 4.0.3. [52] Within the barplots each bar represents one individual felid and the color of the bar represents the % of membership (*Q*) the individual belongs to different genetic clusters.

For pumas, we identified one genetic cluster using the admixture model with correlated allele frequencies (Fig 4A, S2A Fig) and two genetic clusters using the LOCPRIOR model in STRUCTURE, separating most individuals (77%) detected within the northern sampling sites (*Q* > 0.7) into a northern cluster with one disperser (*Q* = 0.17) and two admixed individuals (*Q* = 0.46 and 0.49) (Fig 4B, S2B Fig). Individuals detected across the remaining regions (north-central, central, south-central and south) of Belize were assigned to a southern cluster (mean *Q* = 0.96, range 0.34–1) with the exception of one admixed individual. Using spatially-explicit Bayesian clustering in GENELAND, we identified four genetic clusters and one ghost population for pumas, corresponding to our main *a priori* geographical regions within Belize, including the north (100% residents with mean *Q* = 1), north-central (79% residents with mean *Q* = 1; four migrants with mean *Q* = 0.02), central and central-south (71% residents with mean *Q* = 1; two migrants with mean *Q* = 0.07; three admixed genotypes) and south (80% residents with mean *Q* = 0.98; one migrant with *Q* = 0.24) (Fig 4C).

**Fig 5:**
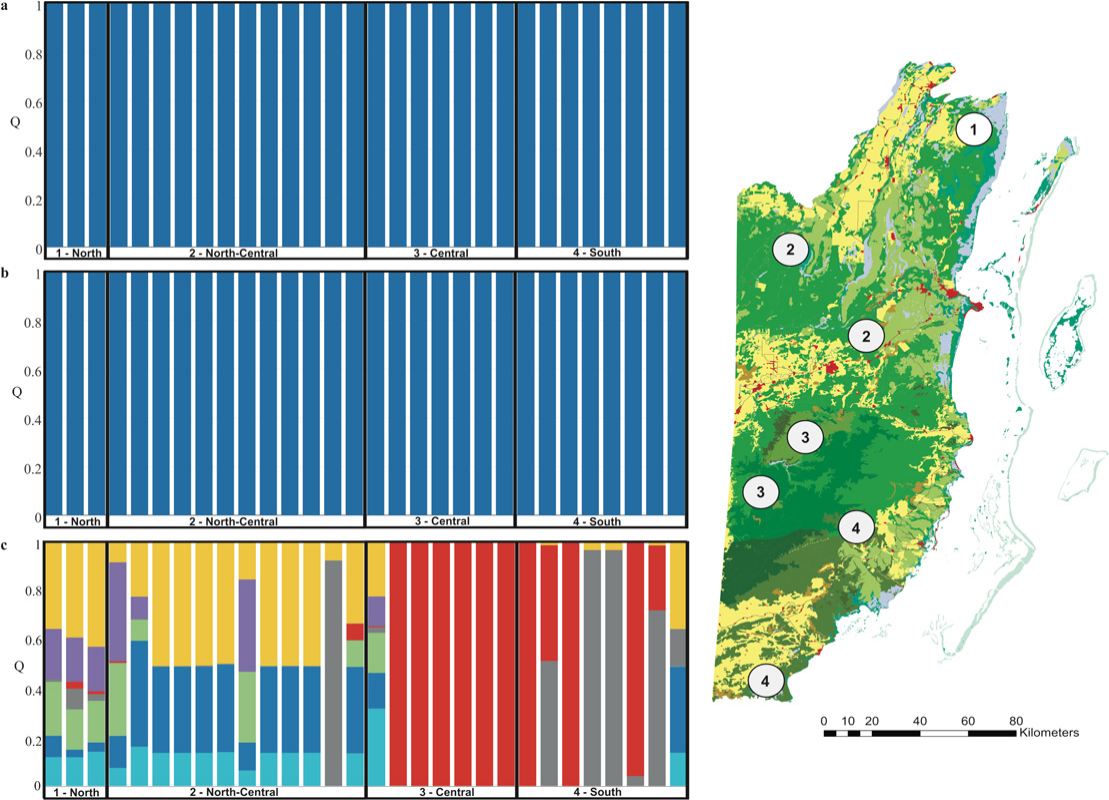
Genetic structure of *Leopardus pardalis* in Belize. Inference for number of genetic clusters (*K*) was based on mean log likelihood LnP(D), using the admixture model with correlated allele frequencies without (a) and (b) with prior sampling location information obtained in STRUCTURE, version 2.3.3 [48], and (c) spatially-explicit Bayesian clustering assignment in GENELAND, version 4.0.3. [52]. Within the barplots each bar represents one individual felid and the color of the bar represents the % of membership (*Q*) the individual belongs to different genetic clusters.

For ocelots, Bayesian clustering analysis in STRUCTURE (admixture and LOCPRIOR models) did not reveal any population subdivision (*K* = 1) (Fig 5A and 5B, S3 Fig). Spatially-explicit Bayesian clustering in GENELAND revealed seven genetic clusters, through which individuals were roughly grouped into two main genetic clusters (northern and southern Belize) (Fig 5C). While the northern cluster showed a high degree of admixture, the southern cluster consisted of several individuals detected at Cockscomb Basin Wildlife Sanctuary that were strongly assigned (*Q* > 0.95) to this site.

**Fig 6:**
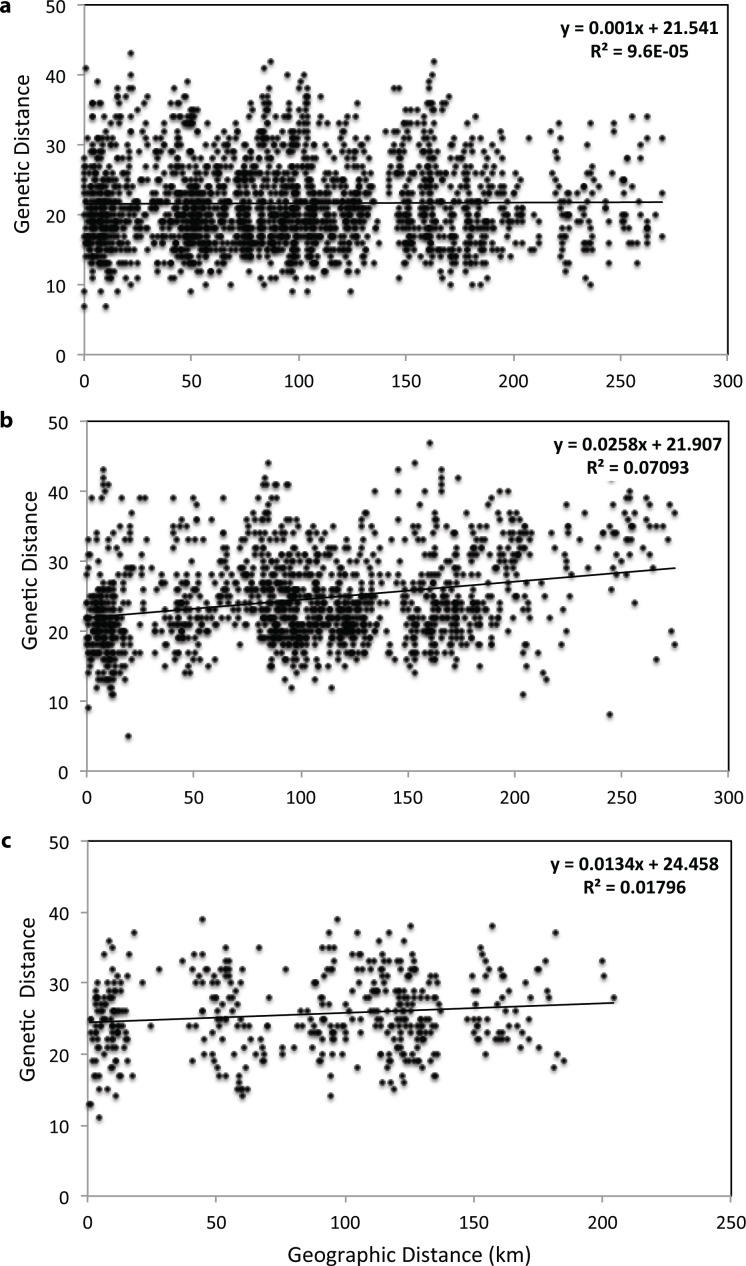
Isolation by distance for Neotropical felids in Belize. Isolation by distance patterns in (a) *Panthera onca*, (b) *Puma concolor*, and (c) *Leopardus pardalis* were assessed by plotting pairwise codominant genotypic distance calculated in GenAlEx, version 6.41 [37] versus pairwise geographic distances (km). Statistical significance was assessed using simple Mantel tests in *ecodist*, version 1.2.9 [56] in R, version 3.1.3 [39]. Each point represents a pairwise comparison among individual felids.

Examination of IBD patterns in jaguars showed no significant correlation (all jaguars, *r* = 0.010, *P* = 0.510, Fig 6A; males, *r* = 0.099, *P* = 0.100; females, *r* = -0.201, *P* = 0.200) between-individual genetic and geographic distances, verifying that geographic distance is not driving genetic differentiation at this spatial scale. Significantly positive relationships between genetic and geographic distance were detected for pumas (Fig 6B), which were most pronounced in females (all pumas, *r* = 0.266, *P* = 0.010; males, *r* = 0.116, *P* = 0.050; females, *r* = 0.379, *P* = 0.010). This suggested that gene flow in female pumas is spatially limited and may be one of the factors driving genetic differentiation in pumas in Belize. In ocelots, we found a significant, but weak signature of IBD (all ocelots, *r* = 0.134, *P* = 0.020, Fig 6C; males, *r* = -0.029, *P* = 0.590; females, *r* = 0.131, *P* = 0.070), indicating that geographic distance potentially has a small effect on gene flow. However, sample sizes in female jaguars and male ocelots were low (> 10 individuals), thus careful interpretation of results is essential.

To examine the spatial extent of genetic structure, we conducted spatial autocorrelation analysis in all three target species and by sex. Female jaguars and male ocelots were not analyzed as individual groups due to low sample sizes. The autocorrelogram for all jaguars showed significantly positive autocorrelation in the first three distance classes (5 km, *r* = 0.050, *P* = 0.002; 10 km, *r* = 0.053, *P* = 0.001; 15 km, *r* = 0.042, *P* = 0.012) and an x-intercept of *r* at ~ 20 km (S4A Fig), empirically confirming the presence of nonrandom spatial structuring and genetic association among individuals at distances < 20 km. Individuals below this threshold, share a higher proportion of genes, than spatially distant individuals. Patterns of spatial autocorrelation detected in male jaguars (S4B Fig), which represent the majority of jaguar samples (88%), were overall consistent with the results for all jaguar samples. The x-intercept of *r* in male jaguars was at 22 km, suggesting a slightly larger spatial extent of autocorrelation in males than the overall sample. In pumas, we also found significantly positive *r*-values for all samples in several close-distance classes (5 km, *r* = 0.046, *P* = 0.014; 10 km, *r* = 0.044, *P* = 0.006; 15 km, *r* = 0.109, *P* = 0.006; 50 km, *r* = 0.048, *P* = 0.001) and an x-intercept at ~ 68 km (S5A Fig). When autocorrelation analysis was conducted separately for both sexes, we detected genetic structure at finer geographic scales in female pumas with positive *r*-values in the first three distance classes, which were significant within the 5 km distance class (*r* = 0.133, *P* = 0.002), and an x-intercept of *r* at ~ 23 km (S5C Fig). In male pumas, spatial autocorrelation was not detected across all distance classes (S5B Fig), suggesting the absence of spatial structure at this geographic scale. The variation in spatial autocorrelation patterns between the sexes in pumas indicated female philopatry and sex-biased dispersal in males. In ocelots, positive autocorrelation was detected for all samples (*r* = 0.110, *P* < 0.000, S6A Fig) and in females (*r* = 0.131, *P* < 0.000, S6B Fig) within the 5 km distance class. The x-intercept of *r* was at ~ 84 km for all samples and at ~ 83 km for females, indicating an overall larger spatial extent of positive autocorrelation in ocelots than in the two larger felids.

Relatedness among sites was significantly different (Kruskal-Wallis rank-sum test, *H* = 38.17, *P* < 0.000) among the three target species, with highest mean relatedness coefficients for jaguars (0.07 ± 0.11, SD), followed by pumas (0.05 ± 0.09, SD), and ocelots (0.03 ± 0.07, SD). Mean relatedness coefficients within sites were slightly higher for jaguars (0.11 ± 0.15, SD), followed by pumas (0.10 ± 0.14, SD), and ocelots (0.08 ± 0.13, SD). However, mean relatedness coefficients within sites were not significantly different among species (Kruskal-Wallis rank-sum test, *H* = 3.38, *P* = 0.184). In all three species, within-site relatedness was significantly higher than among-site relatedness (jaguars [Wilcoxon rank-sum test, *W* = 24726, *P* = 0.024], pumas [Wilcoxon rank-sum test, *W* = 8127, *P* < 0.000], ocelots [Wilcoxon rank-sum test, *W* = 2180, *P* < 0.000]). However, a skewed sex-ratio towards males in jaguars and females in ocelots may introduce bias into the analysis.

Using GENECLASS, we identified three first-generation migrants (i.e. dispersers) for jaguars. The three animals were located, and originally came from, respectively: (1) the central from the south, (2) the central-south from the south, and (3) the south from the north sites. For pumas, we detected a total of four first-generation migrants. The animals were located, and originally came from respectively: (1) north from the north-central, (2) north-central from the north, (3) central from the north, and (4) south from the south-central. For ocelots, we identified two first-generation migrants. One ocelot was located in the northern cluster (Fireburn Nature Reserve) and genetically assigned to the southern cluster. The other ocelot originally came from the southern cluster (Cockscomb Basin Wildlife Sanctuary) and was signed to the northern cluster (Table 5). All first-generation migrants detected in this study were males.

**Table 5:** First-generation migrant analysis indicating dispersers for *Panthera onca*, *Puma concolor*, *and Leopardus pardalis* in Belize.

***Panthera onca***										
** **	** **	** **	**GENECLASS *F***_***0***_ **migrant**		** **	** **	** **	**-log(L) by region**	** **	** **
**Sample ID**	**Sex**	**Origin**	**log(L_home/L_max)**	**A**	**Probability**	**1**	**2**	**3**	**4**	**5**
Jaguar01	M	3	4.43	5	0.007	15.48	12.59	16.65	13.89	12.22
Jaguar02	M	4	4.72	5	0.002	13.81	11.74	11.17	15.55	10.84
Jaguar03	M	5	5.24	1	0.004	8.69	11.72	10.49	11.87	13.92
***Puma concolor***										
** **	** **	** **	**GENECLASS *F***_***0***_ **migrant**		** **	** **	** **	**-log(L) by region**	** **	** **
**Sample ID**	**Sex**	**Origin**	**log(L_home/L_max)**	**A**	**Probability**	**1**	**2**	**3**	**4**	**5**
Puma01	M	1	6.59	2	0.001	19.27	12.68	16.23	12.75	18.29
Puma02	M	2	3.22	1	0.008	12.15	15.37	13.37	12.39	15.21
Puma03	M	3	2.53	1	0.008	12.66	18.27	15.19	17.26	23.97
Puma04	M	5	4.83	4	0.000	14.37	14.2	12.95	9.67	14.49
***Leopardus pardalis ***		*** ***								
** **	** **	** **	**GENECLASS *F***_***0***_ **migrant**		** **	** **	** **	**-log(L) by region**	** **	** **
**Sample ID**	**Sex**	**Origin**	**log(L_home/L_max)**	**A**	**Probability**		**1**	** **	**2**	** **
Ocelot01	M	1	0.94	2	0.009		17.87		16.93	
Ocelot02	M	2	3.17	1	0.001		10.07		13.25	

First-generation migrants (*F*_*0*_) were identified across five geographical regions (1 north, 2 north-central, 3 central, 4 south-central, 5 south) using likelihood computations (L_ home/L_max) in GENECLASS, version 2.0 [59]. For *Leopardus pardalis*, we identified *F*_*0*_ migrants across two general regions (1 north, 2 south) in Belize. Origin, geographical sampling location of individual felid; A, geographical location based on genetic assignment of individual felid.

**Fig 7:**
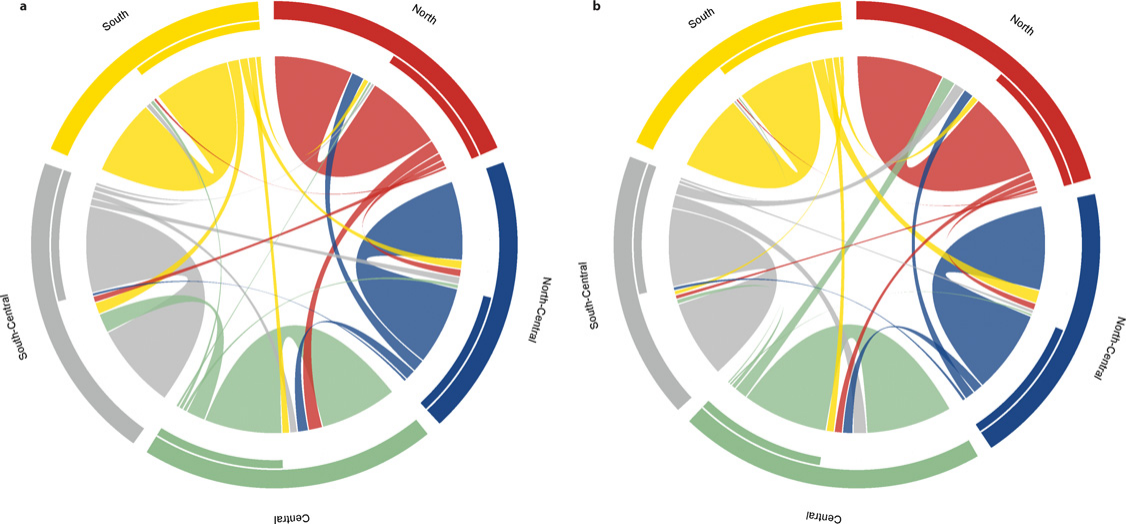
Contemporary migration rates for *Panthera onca* and *Puma concolor* in Belize. Circular plots of contemporary bidirectional migration rates in (a) *Panthera onca* and (b) *Puma concolor* between and within five geographical regions (north, north-central, central, south-central, south) in Belize derived by BayesAss+, version 3.0 [62].

Contemporary and bidirectional migration rates estimated between all sampling sites in BayesAss+ suggested an average migration rate of 0.07 (range 0.02–0.18) for jaguars across Belize (Fig 7A, Table 6). Migration rates, which represent the proportion of individuals that move from corresponding source population each generation, were highest between the central and south-central sites (0.18). Migration rates for pumas were similar (mean = 0.06, range 0.01–0.12; Fig 7B) to jaguars across Belize, with the highest estimates between central and north, south-central and central, and south and north-central sites (0.12). Mean migration rates for ocelots were relatively high (0.15) and two-to-three times higher from the southern to the northern site (0.21) than the other way around (0.09). Asymmetrical migration rates were detected among a few sites for jaguars (central and south-central) and pumas (north-central and central, north-central and south, central and south-central), with all other site comparisons suggesting symmetrical migration as indicated by overlapping 95% confidence intervals (Fig 7, Table 6).

**Table 6:** Contemporary migration rates for *Panthera onca*, *Puma concolor*, and *Leopardus pardalis* in Belize.

	**Migration from**						** **		** **						
*** Panthera onca***	**North**		** **			**North-Central**			**Central**		**South-Central**			**South**	** **
**Migration into**	***m***		**95% CI**			***m***		**95% CI**	***m***	**95% CI**	***m***	**95% CI**		***m***	**95% CI**
**North**	0.72		0.04			0.12		0.06	0.03	0.03	0.03	0.02		0.05	0.04
**North-Central**	0.07		0.05			0.72		0.05	0.04	0.04	0.07	0.04		0.08	0.05
**Central**	0.13		0.07			0.1		0.07	0.71	0.03	0.07	0.05		0.07	0.05
**South-Central**	0.06		0.05			0.03		0.03	0.18	0.05	0.79	0.05		0.11	0.06
**South**	0.03		0.03			0.02		0.03	0.04	0.02	0.05	0.03		0.7	0.03
	**Migration from**							** **	** **						
*** Puma concolor***	**North**	** **			**North-Central**				**Central**		**South-Central**			**South**	** **
**Migration into**	***m***	**95% CI**			***m***		**95% CI**		***m***	**95% CI**	***m***		**95% CI**	***m***	**95% CI**
**North**	0.8	0.06			0.09		0.04		0.12	0.03	0.1		0.03	0.06	0.04
**North-Central**	0.06	0.05			0.76		0.06		0.03	0.02	0.03		0.03	0.12	0.07
**Central**	0.07	0.04			0.09		0.03		0.78	0.03	0.12		0.04	0.07	0.05
**South-Central**	0.04	0.03			0.03		0.02		0.05	0.02	0.71		0.03	0.04	0.04
**South**	0.02	0.02			0.01		0.01		0.02	0.02	0.03		0.03	0.71	0.03
	**Migration from**														
*** Leopardus pardalis***	** **	**North**		** **			** **		** **	**South**	** **		** **	** **	
**Migration into**	*** ***	***m***		**95% CI**			** **		*** ***	***m***	**95% CI**		** **		
**North**		0.79		0.09						0.09	0.06				
**South**		0.21		0.09						0.91	0.06				

Contemporary and bi-directional migration rates were assessed among five geographical regions (north, north-central, central, south-central, south) within Belize using BayesAss+, version 3.0 [62]. Migration rates equal the proportion of individual felids that are derived from a corresponding source population each generation. *m*, migration rate; 95% CI, confidence intervals for migration rates.

## Discussion

Preserving and restoring genetic connectivity in anthropogenically altered and fragmented landscapes is a key aspect of conservation (e.g., [5, 64]). Comparative conservation genetics studies increase our understanding of species-specific responses to habitat loss and fragmentation, and have been invaluable for developing conservation and management strategies for multiple species of conservation concern (e.g., [65, 66–70]). The main objective of our study was to use noninvasive genetic sampling to examine levels of genetic diversity, population structure, and contemporary gene flow of multiple elusive felid species living in fragmented tropical forest habitats. Ultimately, we aim to support current and future conservation and management efforts for wild felids within Belize and throughout their range. Our results provide the first comparative genetic assessment of wild populations of jaguars, pumas, and ocelots within Mesoamerica. The use of scat detector dogs to increase detection rates of fecal samples and the application of standardized methods for sample collection and storage of fecal DNA [35] have been instrumental in successfully applying these methods on multiple felid species in tropical environments, where relatively little is known about genetic diversity and structure of these species.

### Genetic diversity of three Neotropical felids

Genetic diversity across Belize was moderate for all species and lowest for jaguars, followed by pumas and ocelots (Table 2), which likely reflects differences in effective population sizes for the three species. While there were no significant differences in diversity levels among regions for all three target species, levels were lowest in more remote and isolated areas of Belize, including the far north for jaguars (*H*_*e*_ = 0.54), the south (*H*_*e*_ = 0.44) for pumas, and northern Belize for ocelots (*H*_*e*_ = 0.61). In comparison with other genetic studies, using *Fca* microsatellite markers [71], levels of genetic diversity for Belizean jaguars were relatively low. For example, Brazilian (*H*_*e*_ = 0.73) [25] and Colombian jaguars (*H*_*e*_ = 0.85) [23] were genetically more diverse. But genetic diversity estimates for Central American jaguars obtained through a range-wide genetic study [24] were concordant with our findings. For pumas, diversity estimates in Belize (*H*_*e*_ = 0.65) were similar [72] or higher [73, 74] compared to North American pumas and lower than South American pumas (21, 27) using *Fca* microsatellite markers [71]. Culver, Johnson [75] observed similar levels of genetic diversity for pumas in Central America. Belizean ocelots had lower levels of genetic diversity compared to South American populations (e.g., [21, 76]), but showed higher estimates than ocelots studied in northeastern Mexico and southern parts of the United States [77, 78]. The differences in genetic diversity estimates for Central and South American felids can be explained by a combination of historical and contemporary factors, including range contraction/expansion, and changes in population size and gene flow (e.g., [24, 75, 77, 79]). Nonetheless, a strict direct comparison of diversity measures between these studies should be viewed cautiously since different sets of *Fca* microsatellite markers were used by most studies.

### Fine-scale genetic structure and contemporary gene flow

Using a combination of individual- and population-based genetic structure analyses revealed low to moderate levels of genetic differentiation for Neotropical felids within Belize, with jaguars showing the lowest levels of genetic subdivision (i.e. highest genetic connectivity) across the country, followed by ocelots and pumas. Considering the dispersal capabilities, particularly of the two larger felids (e.g., [9, 33]), the presence of large tracts of forests, and the relatively small total area of Belize (~ 22,966 km^2^; Fig 1), we were not surprised to see relatively high levels of gene flow and low genetic differentiation. However, our fine scale genetic analyses revealed that jaguars in Belize, may not exist in total panmixia, but rather exhibit low levels of genetic differentiation when genetic divergence was estimated indirectly (*F* statistics, AMOVA, DAPC) or through non-spatial Bayesian clustering methods. Spatial autocorrelation analysis detected genetic structuring in jaguars at fine geographic scale (< 20 km) across all samples and in males (females were not analyzed separately due to small sample size). This indicated high genetic associations among individuals sampled in close proximity, which could be caused by several factors including natal philopatry, habitat fragmentation, and/or clustered and high-density sampling efforts (e.g., [74, 80]). Since male philopatry in jaguars is unlikely, spatial autocorrelation detected at close distance classes was either a sampling artifact or caused by limited gene flow resulting from restricted movement of individuals across the landscape as described in other studies (e.g., [81, 82]). When using spatially informed Bayesian clustering, we detected moderate levels of genetic differentiation. Spatial Bayesian clustering methods, which integrate geographically explicit prior distributions into the inference process, have been shown to be more powerful in detecting weak genetic discontinuities in small data sets (e.g., [66, 72, 83]). However, careful consideration is necessary when interpreting the Bayesian clustering results, since opportunistic sampling, as well as sampling density, may cause inconsistencies in the clustering analysis [77, 78]. Multiple methods roughly grouped jaguars into a northern (north and north-central sites) and a southern (central, central-south, south sites) cluster, which are separated by a ~ 50 km wide stretch of open and human-dominated landscape bordering north-central and central Belize. Our results suggested that the genetic subdivision detected corresponded largely to patterns of habitat fragmentation and human disturbance. This finding was concordant with another genetic study on jaguars showing that human-dominated landscapes have the potential to limit gene flow in jaguars on a fine geographic scale [25]. Although jaguars are considered generalist species and utilize a variety of habitat types (e.g., [1, 9, 84, 85]), they prefer areas with tree cover [86], close to water bodies [11], and are less likely to use human-modified landscapes such as agricultural [87] and more populated areas [88]. Former studies also showed that physical barriers such as roads limit jaguar movement, with females exhibiting a high degree of road avoidance [88, 89]. However, spatial Bayesian clustering analysis also identified several admixed individuals (Fig 3C), which indicates that jaguars did not only move across the region, but were also able to reproduce. In addition, our contemporary gene flow analysis found evidence for recent dispersal events by classifying three male jaguars as first-generation migrants. Two of the jaguars were detected in central (Chiquibul Forest Reserve and National Park) and south-central (Cockscomb Basin Wildlife Sanctuary) Belize, and were genetically assigned to southern Belize. One male jaguar that was originally sampled in the Golden Stream Corridor Preserve in southern Belize, was genetically assigned to the most northern site, which is located approximately 250 km away. These findings were supported by contemporary migration rates, which suggest that movement of jaguars between some sites within Belize is relatively common, although asymmetric migration rates were detected in some cases (Fig 7A, Table 6). Interestingly, we detected asymmetric migration rates within central Belize (Chiquibul Forest Reserve and National Park, Mountain Pine Ridge Forest Reserve) to Cockscomb Basin Wildlife Sanctuary, suggesting that about 18% of the jaguars moved out from these areas during this generation, whereas immigration rates into these areas were not higher than 7% (Fig 7A). Central Belize, including the Chiquibul Forest Reserve and other areas close to the Guatemalan border (e.g., Columbia River Forest Reserve), have experienced high rates of incursions by illegal Guatemalan immigrants during the last forty years (e.g., [31, 90, 91]). Illegal activities within these protected areas include farming, hunting, harvesting of timber and non-timber products, and drug trafficking. In addition, industrial activities such as agroindustry, hydroelectricity, mining, logging, and road expansions into more remote areas have increased the rates of forest loss and human impact [90]. The elevated levels of human disturbance and land use change within these protected forest habitats could potentially cause emigration rates to increase for jaguars. In contrast, outward migration rates from Cockscomb Basin Wildlife Sanctuary, which represents undisturbed forest habitat and is also part of the Maya Mountain Massif, a large block of tropical forest in Belize, are relatively low (5% to south, 3% to north, 7% to central, and 7% to north-central Belize). Besides human-induced impacts (e.g., human developments, hunting, human-wildlife conflict), migration rates may be also driven by ecological (e.g., population density, competition, social system, prey density), and/or behavioral (e.g., dispersal, habitat use) factors as observed in several other carnivore studies (e.g., [67, 72, 75, 92–95]). Jaguars sampled at the Rio Bravo Conservation Management Area, showed relatively high outward migration rates towards northern and central Belize (Fig 7A). This could be explained by Rio Bravo Conservation Management Area being part of La Selva Maya, which likely represents one of the most important source populations for jaguars within the region, potentially exhibiting dispersal induced by positive density-dependence. In conclusion, our results suggested that jaguars within Belize are genetically still relatively well connected; nonetheless, there is fine-scale genetic differentiation occurring and if habitat loss and fragmentation continue at the current rate, jaguar populations may consequently exhibit much stronger genetic structure, as shown in a previous fine-scale study [25].

For pumas, which are found in a wide range of natural environments (e.g., [1, 9, 96]), we detected moderate levels of genetic differentiation within Belize with sub-structuring between most sites surveyed. Spatial, individual-based Bayesian clustering analysis showed that genetic clusters are more distinct and less admixed in areas experiencing higher levels of human disturbance. Genetic subdivision was most pronounced between Fireburn Nature Reserve, one of the geographically most isolated protected areas in northern Belize, and all other areas surveyed. These findings matched with previous fine-scale genetic studies of pumas (e.g., [1, 9]), which concluded that despite the pumas’ ability to disperse over long distances [72, 97–100], genetic substructure increases in areas where habitat is less contiguous and is altered due to anthropogenic impacts (e.g., [33, 101]), or is negatively influenced by habitat barriers such as rivers [97], open deserts and grasslands [98]. However, the detection of admixed individuals and dispersers suggested that pumas were able to move across Belize and reproduce to some extent. Protected areas in central (Chiquibul Forest Reserve and National Park, Mountain Pine Ridge Forest Reserve) and south-central (Cockscomb Basin Wildlife Sanctuary) Belize formed one genetic cluster with several admixed individuals and dispersers from northern Belize (Fig 4C). Furthermore, our study also showed evidence for fine-scale genetic structure in female pumas, inferring that females living in close geographic proximity are on average genetically closely related, exhibiting short dispersal distances and female philopatry as described by other puma studies [102, 103] and in mammals in general (e.g., [104, 105]). In contrast, and different from jaguars, male pumas did not show evidence of positive spatial autocorrelation at any geographic distance tested, suggesting that dispersal in pumas across this landscape is male-biased, which is consistent with previous studies [106, 107]. However, the spatial extent of positive autocorrelation described by our study was smaller in comparison to North American pumas (e.g., [74, 106, 108]), suggesting that dispersal movements in Neotropical pumas, especially in females may be more restricted. Furthermore, contemporary gene flow analysis found evidence for four male first-generation migrants (i.e. dispersers) in Belizean pumas. We detected two dispersers between north-central and the most northern site. One puma was detected in the Mountain Pine Ridge Forest Reserve in central Belize and genetically assigned to the most northern sites within Belize. The fourth migrant was detected in the Golden Stream Corridor Preserve in southern Belize, and originated from the neighboring Cockscomb Wildlife Basin Sanctuary in south-central Belize. Our results suggested that most dispersal events were directional and that dispersers moved away from either the most northern or the most southern sites, which both overlap with areas experiencing high levels of deforestation and land conversion (e.g., [31, 109]). Contemporary migration rate analysis, which suggested that on average about 6% of Belizean pumas move among different geographical areas, complemented these findings. An interesting observation was that outward migration rates for pumas from the Cockscomb Basin Wildlife Sanctuary moving into central Belize (Chiquibul Forest Reserve and National Park, Mountain Pine Ridge Forest Reserve) were more than double in comparison to their inward migration rates, which stands in contrast to our findings for jaguars. Despite the fact that both areas are separated by the Maya Mountain range, we believe that high population densities for jaguars and corresponding levels of interspecific competition at Cockscomb Basin Wildlife Sanctuary may cause pumas to have a two to three times higher outward migration rate in comparison to levels of incoming pumas. Furthermore, Rio Bravo Conservation Management Area showed moderate puma outward migration rates (9%) into central Belize, whereas inward migration was only one third of it. Similar to jaguars, Rio Bravo Conservation Management Area may also represent an important source population for pumas within the region. Migration rates into the most southern sites were generally limited (≤ 3%), which most likely was caused by higher levels of land conversion and human disturbance in this region. Despite the detection of several first-generation migrants within the country, we conclude that pumas exhibited moderate levels of genetic differentiation within Belize, which may be primarily driven by spatial autocorrelation patterns in female pumas.

For ocelots, non-spatial Bayesian clustering analysis rendered support for no or low levels of genetic differentiation. Furthermore, autocorrelation analysis suggested a large spatial extent of genetic structure (~ 84 km) in a mesocarnivore, indicating that gene flow for ocelots across this landscape may be relatively high. Given ocelots’ smaller body size and their tolerance to human activity to some extent (e.g., [13]) they may be more successful moving through human-dominated landscapes than the two larger felids. However, spatial autocorrelation analysis also revealed fine-scale genetic structure over short distances (< 5 km) in female ocelots, suggesting female philopatry. In addition, spatial Bayesian clustering resulted in moderate genetic subdivision, roughly grouping Belizean ocelots into a northern and southern cluster, indicating that human-dominated areas bordering north-central and central Belize presumably restrict gene flow between these sites, but still allow for occasional dispersal events. From previous studies it is known that ocelots are habitat specialists preferring closed forest types and dense thorn scrubs (e.g., [9, 96, 110, 111]), thus movement across central Belize may be limited by open areas (e.g., savannah, agricultural areas). Nonetheless, among the northern and southern cluster, we documented movement into and out of both areas. First, we observed two male first-generation migrants in ocelots going from the north to the south and one from south to north (Table 5). Second, migration rates between northern and southern sites were also found to be directional, with higher levels of gene flow going from the north to the south (21% of individuals for this generation), than from southern to northern regions (9%) (Fig 7; Table 6). Although ocelots are still considered widely abundant across Central America, they are understudied, and we found that the effects of habitat loss and fragmentation may have the potential to severely reduce genetic connectivity, which has been documented in ocelots at the northern extent of their range (e.g., [78]) and other small felid populations (e.g., [112, 113]).

### Comparative genetic structure of two sympatric felids

This study directly compared patterns of fine-scale genetic connectivity in multiple Neotropical felids. For jaguars and pumas, which differ in size across regions of coexistence, jaguars are the larger, dominating species [114]. We observed regional differences in genetic connectivity levels within Belize, with a tendency for pumas to be less connected than jaguars. We did not include ocelots in this section due to the smaller sample size obtained for this species. Our hypothesis that pumas would experience higher levels of genetic connectivity relative to sympatric jaguars was not supported. Varying levels of genetic differentiation for jaguars and pumas were especially evident between the most northern and most southern sites within Belize, where felids are likely confined to small and fragmented forest patches surrounded by less suitable habitat and human-modified areas of higher disturbance (e.g., human-wildlife conflict, farming). This pattern of genetic divergence was more pronounced in pumas. However, we also detected evidence for first-generation migrants in both species (3 dispersers in jaguars, 4 dispersers in pumas). Considering that this analysis depends on sample coverage and several other factors, the results only represent an approximation of the actual dispersal events. We found a general trend that pumas were more likely to disperse to geographically close or neighboring areas in comparison to jaguars. Comparative relatedness analysis supported the findings related to genetic divergence by revealing that jaguars had significantly higher pairwise relatedness values among sites than pumas, and showed a generally higher tendency to disperse from their natal areas. Autocorrelation analysis revealed significant genetic structure in jaguars and female pumas at close distance classes with positive correlations almost three times as high in female pumas. Consequently, we reject the null hypothesis of a random distribution of genotypes at this spatial scale. The spatial structuring we detected can be associated with a multitude of species-specific processes, including social and mating systems, sex-biased dispersal, and/or restricted movement and consequently gene flow (e.g., [57, 67]). Although it is difficult to differentiate between the effects of social organization and reduced gene flow, differences in autocorrelation patterns between sexes in pumas indicated that female philopatry and male-biased dispersal may be primarily driving the spatial structure detected, which is common in felids and mammals in general (e.g., [9, 104]). Alternatively, for jaguars, positive spatial structure at close geographic distances, especially for males, may be the result of restricted gene flow across the landscape, which stands in contrast to our findings for male pumas. In addition, the spatial extent of genetic structure detected in jaguars and pumas (with the exception of male pumas) also suggested that effective dispersal distances across this heterogeneous landscape are potentially limited.

Gene flow may be also driven by various other ecological factors, including the degree to which habitat use and human disturbance limit movement in wide-ranging species such as jaguars and pumas. Former studies described pumas as opportunistic in their habitat use since they are known to use a wider variety of habitats in comparison to jaguars (e.g., [9, 96]). In contrast to jaguars, which have a tendency to avoid open areas and prefer dense forest habitats, previous studies also reported movement of pumas through disturbed and human-developed areas (e.g., [25, 72, 97, 98, 115]). Opposite to these findings and in agreement with our study, research on habitat use of jaguars and pumas in Belize concluded that pumas were less likely to be found outside of protected areas than jaguars due to a differential tolerance to human disturbance and resource limitation [116]. Davis, Kelly [117] also reported that pumas, and jaguars to a lesser extent, were sensitive to human disturbance, even within protected areas of Belize. In addition, interspecific spatial interactions between sympatric jaguars and pumas resulting in spatial avoidance of jaguars by pumas as described by a few studies (e.g., [118, 119]), may also affect movement and consequently gene flow patterns of these two sympatric species across the landscape.

### Conservation and management implications

With increasing anthropogenic impact and landscape change, it is crucial to genetically monitor wild felid populations. Understanding levels of fine-scale gene flow and genetic structure patterns of multiple co-occurring felid species reveal behavioral and other factors that influence gene flow across the landscape, which is vital for planning and prioritizing future conservation and management efforts on a countrywide scale. This study provides comprehensive baseline conservation genetic data and demonstrates that noninvasive genetic sampling is an efficient research approach to simultaneously assess levels of genetic diversity and differentiation of multiple Neotropical felid species in the wild. We found that genetic diversity for wild felids in Belize is moderate and that levels of genetic connectivity within the country are moderate to high. Although Belize has a high proportion of forest cover (~ 62.7%) and protected areas (~ 36%) compared to other Mesoamerican countries, we believe that levels of genetic connectivity are likely to decrease if habitat loss and fragmentation continue at the current rate. Despite the dispersal capabilities of the two large felids, our study detected evidence for fine-scale genetic differentiation particularly between northern and southern sites, indicating that the more-developed and human-dominated areas adjacent to north-central and central Belize may be subtlety-affecting movements of felids. Consequently, we recommend prioritizing countrywide conservation and management efforts for wild felids, such as maintaining and enhancing biological connectivity among the national protected area system within Belize through strengthening formerly identified wildlife movement corridors (Northern Belize Corridor, Central Belize Corridor, Southern Belize Corridor) [120, 121]. The fledgling Central Belize Corridor, which connects Belize’s two largest forest blocks (north: RBCMA, Yalbac, Laguna Seca, Gallon Jug; south: Maya Mountain Massif), represents a critical link in the Mesoamerican Biological Corridor within this region, and deserves special attention. In addition, we also recommend conducting continued genetic monitoring efforts, and assessing functional connectivity of movement corridors through a landscape genetics approach. Furthermore, we also encourage large-scale research efforts focusing on multiple, sympatric species to increase understanding of species interactions and responses to fragmented landscapes and to develop regional multi-species conservation and management strategies. With Mesoamerica bearing one of the highest deforestation rates in the world, and lacking in genetic studies of wild felids in general, genetic-based monitoring focusing on wildlife of conservation concern is needed to assess connectivity and genetic health across the entire region.

## Supporting Information

S1 Fig Results of STRUCTURE analysis for *Panthera onca* in Belize. The optimal *K* value for the admixture model (a) without and (b) with adding sampling locations as prior (LOCPRIOR) was chosen based on posterior probability (mean LnP(*K*)) and delta *K* (Δ*K*) for each *K* value.
(DOCX)

S2 Fig Results of STRUCTURE analysis for *Puma concolor* in Belize. The optimal *K* value for the admixture model (a) without and (b) with adding sampling locations as prior (LOCPRIOR) was chosen based on posterior probability (mean LnP(*K*)) and delta *K* (Δ*K*) for each *K* value.
(DOCX)

S3 Fig Results of STRUCTURE analysis for *Leopardus pardalis* in Belize. The optimal *K* value for the admixture model (a) without and (b) with adding sampling locations as prior (LOCPRIOR) was chosen based on posterior probability (mean LnP(*K*)) and delta *K* (Δ*K*) for each *K* value.
(DOCX)

S4 Fig **Spatial autocorrelogram for *Panthera onca* in Belize** (a. all jaguars [*n* = 65]; b. male jaguars [*n* = 57]) showing the genetic correlation coefficient (*r*) as a function of geographic distance across spatial distance classes. Dashed red lines represent upper (U) and lower (L) bounds of the null distribution based on 9,999 random permutations. Error bars represent 95% confidence intervals about *r* based on 999 bootstraps.
(DOCX)

S5 Fig **Spatial autocorrelogram for *Puma concolor* in Belize** (a. all pumas [*n* = 54]; b. male pumas [*n* = 30], c. female pumas [*n* = 24]) showing the genetic correlation coefficient (*r*) as a function of geographic distance across spatial distance classes. Dashed red lines represent upper (U) and lower (L) bounds of the null distribution based on 9,999 random permutations. Error bars represent 95% confidence intervals about *r* based on 999 bootstraps.
(DOCX)

S6 Fig **Spatial autocorrelogram for *Leopardus pardalis* in Belize** (a. all ocelots [*n* = 30]; b. female ocelots [*n* = 25]) showing the genetic correlation coefficient (*r*) as a function of geographic distance across spatial distance classes. Dashed red lines represent upper (U) and lower (L) bounds of the null distribution based on 9,999 random permutations. Error bars represent 95% confidence intervals about *r* based on 999 bootstraps.
(DOCX)

S1 Materials and Methods PCR reactions and thermocycling conditions for multiplex 1–3. (DOCX)
