# Supplementary material for: A Comparative Analysis of Genetic Diversity and Structure in Jaguars (Panthera onca), Pumas (Puma concolor), and Ocelots (Leopardus pardalis) in Fragmented Landscapes of a Critical Mesoamerican Linkage Zone
Source: PLoS One. 2016 Mar 14;11(3):e0151043. doi: 10.1371/journal.pone.0151043 (PMC4790928; doi:10.1371/journal.pone.0151043)

**Figure S3. Results of STRUCTURE analysis for *Leopardus pardalis* in Belize**. The optimal *K* value for the admixture model (a) without and (b) with adding sampling locations as prior (LOCPRIOR) was chosen based on posterior probability (mean LnP(*K*)) and delta *K* (Δ*K*) for each *K* value.

(a)


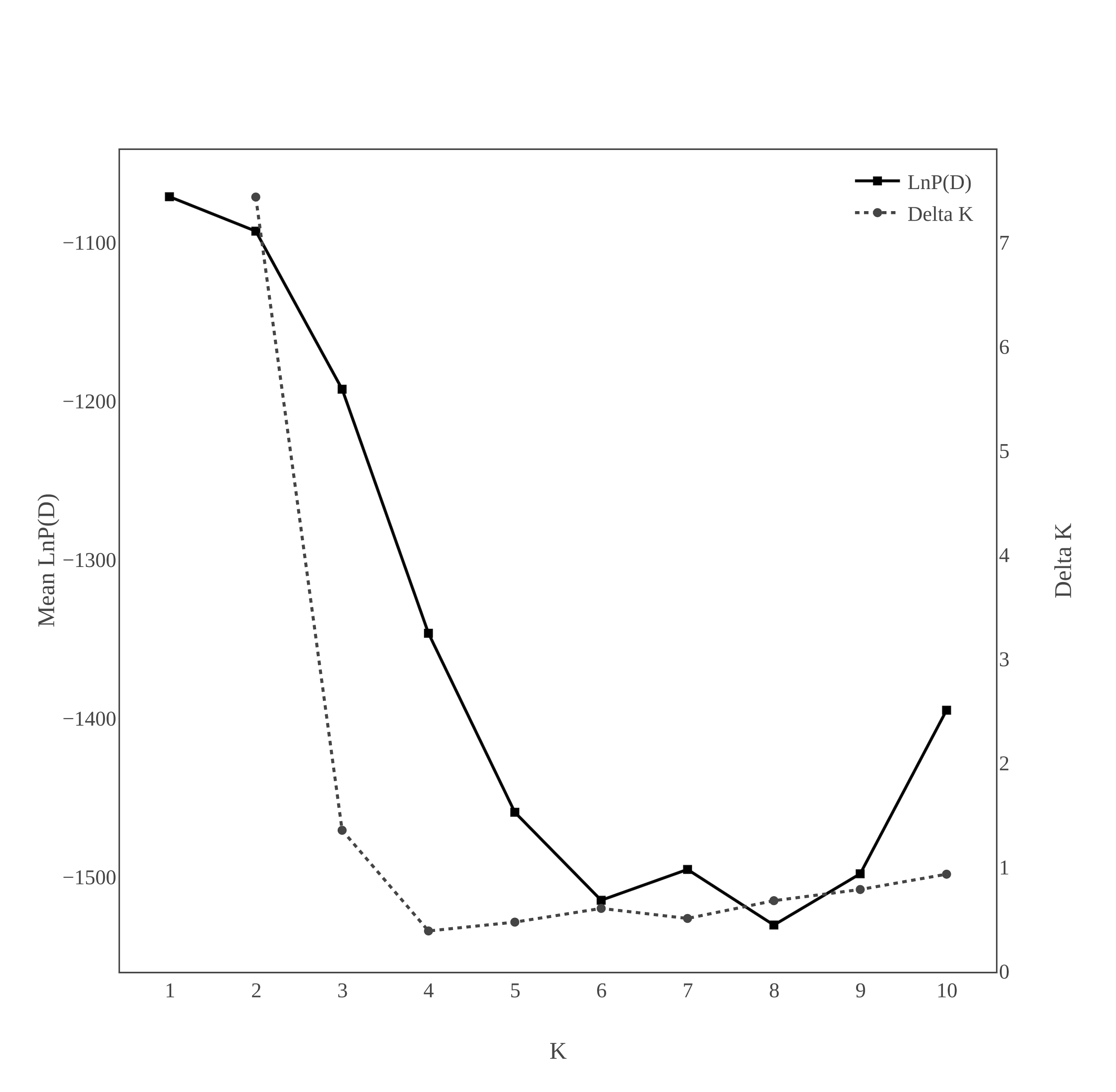


(b)


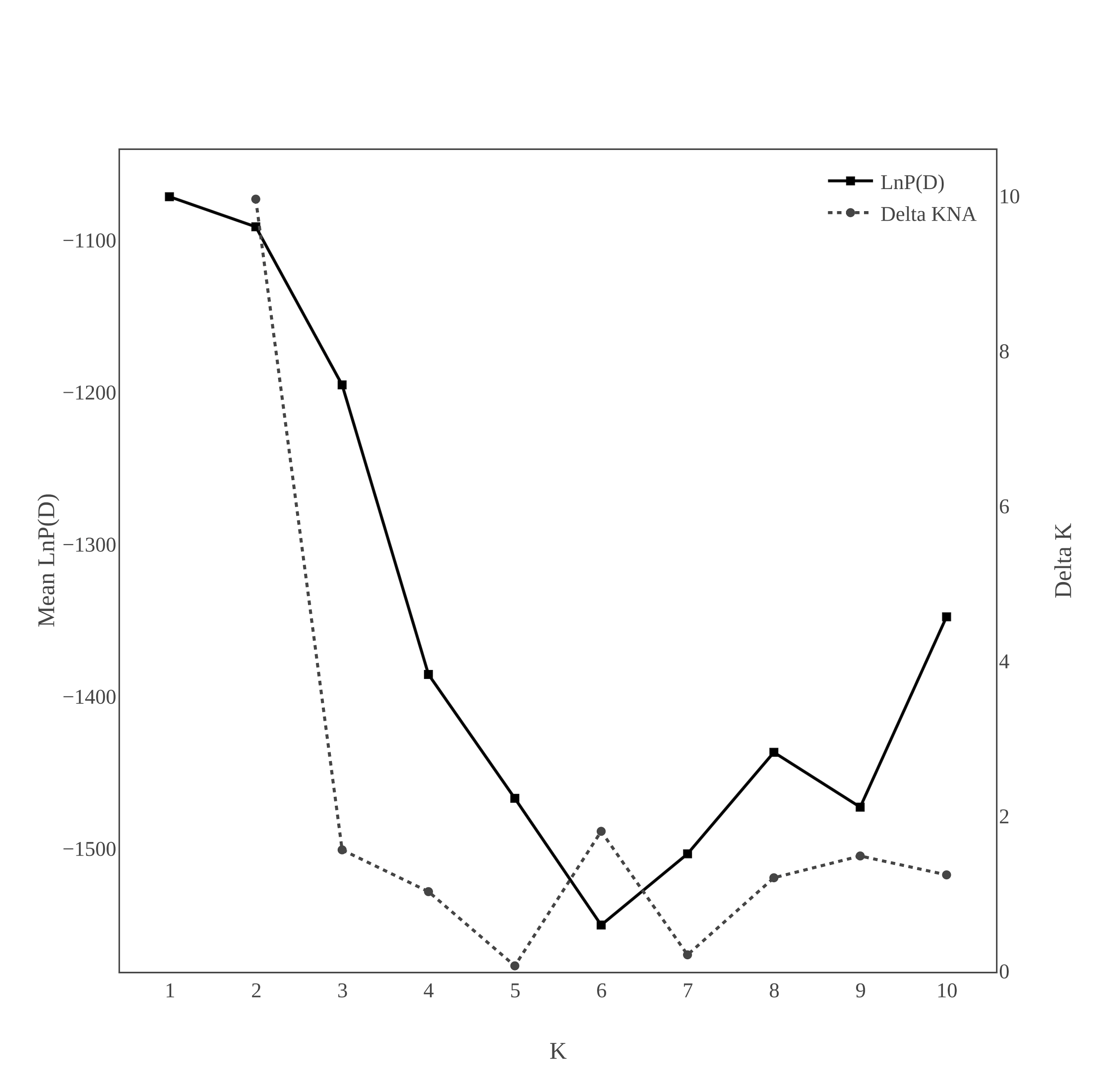

Supplement: S3 Fig — The optimal K value for the admixture model (a) without and (b) with adding sampling locations as prior (LOCPRIOR) was chosen based on posterior probability (mean LnP(K)) and delta K (ΔK) for each K value. (DOCX) [file pone.0151043.s003.docx]
