# Supplementary material for: A Comparative Analysis of Genetic Diversity and Structure in Jaguars (Panthera onca), Pumas (Puma concolor), and Ocelots (Leopardus pardalis) in Fragmented Landscapes of a Critical Mesoamerican Linkage Zone
Source: PLoS One. 2016 Mar 14;11(3):e0151043. doi: 10.1371/journal.pone.0151043 (PMC4790928; doi:10.1371/journal.pone.0151043)

**Figure S5. Spatial autocorrelogram for *Puma concolor* in Belize** (a. all pumas [*n* = 54]; b. male pumas [*n* = 30], c. female pumas [*n* = 24]) showing the genetic correlation coefficient (*r*) as a function of geographic distance across spatial distance classes. Dashed red lines represent upper (U) and lower (L) bounds of the null distribution based on 9,999 random permutations. Error bars represent 95% confidence intervals about *r* based on 999 bootstraps.


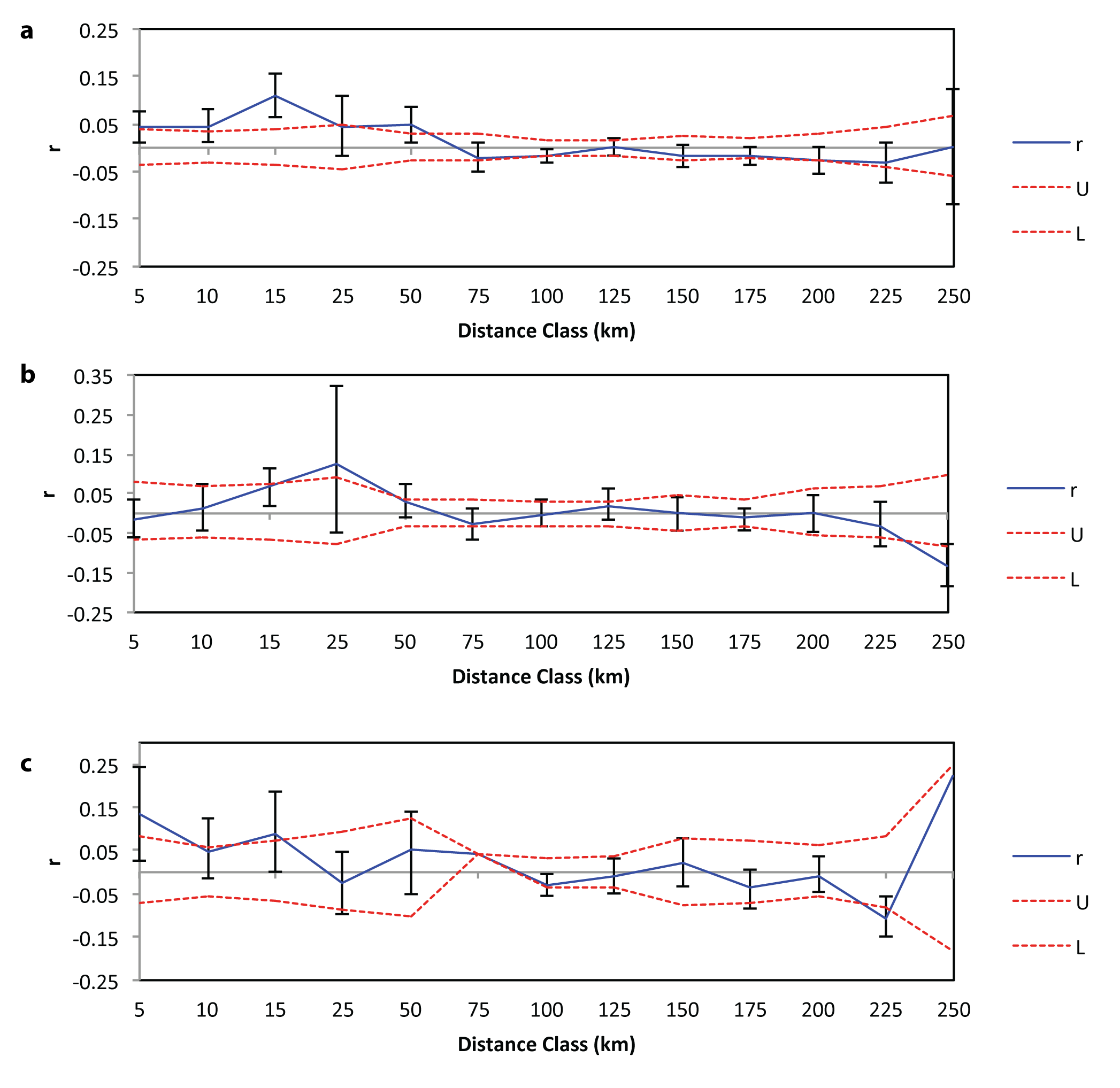

Supplement: S5 Fig — Spatial autocorrelogram for Puma concolor in Belize (a. all pumas [n = 54]; b. male pumas [n = 30], c. female pumas [n = 24]) showing the genetic correlation coefficient (r) as a function of geographic distance across spatial distance classes. Dashed red lines represent upper (U) and lower (L) bounds of the null distribution based on 9,999 random permutations. Error bars represent 95% confidence intervals about r based on 999 bootstraps. (DOCX) [file pone.0151043.s005.docx]
