# Supplementary material for: A Comparative Analysis of Genetic Diversity and Structure in Jaguars (Panthera onca), Pumas (Puma concolor), and Ocelots (Leopardus pardalis) in Fragmented Landscapes of a Critical Mesoamerican Linkage Zone
Source: PLoS One. 2016 Mar 14;11(3):e0151043. doi: 10.1371/journal.pone.0151043 (PMC4790928; doi:10.1371/journal.pone.0151043)
Supplement: S1 Materials and Methods — (DOCX) [file pone.0151043.s007.docx]

**S1 Materials and Methods**. **PCR reactions and thermocycling conditions for multiplex 1 – 3.**

We used 14 highly polymorphic microsatellite loci (1-3) and two DNA markers associated with the Y sex chromosome carried by males (Zn, Zn-finger; Amel, Amelogenin), but not by females (4), labeled with fluorescent dyes and arranged in three PCR multiplex reactions (multiplex 1 - F124-PET, FCA391-NED, FCA043-NED, FCA275-VIC, FCA096-6-FAM, FCA126-PET, FCA090-6-FAM, Zn-6-FAM; multiplex 2 - F85-VIC, F98-6-FAM, FCA741-PET, FCA225-PET, FCA008-6-FAM, Amel-6-FAM; multiplex 3 - F53-NED, FCA441-6-FAM) to enhance performance and efficiency. The three multiplexes each contained 5.2 µL PCR mixture and 1.8 µL of DNA. Multiplex 1 included 3.5 µL 1 x concentrated Qiagen Master Mix (Qiagen, Inc.), 0.7 µL of 0.5 x concentrated Qiagen Q solution (Qiagen, Inc.), 0.98 µL of primers (0.10µM F124, 0.34µM FCA391, 0.07µM FCA043, 0.13µM FCA275, 0.21µM FCA096, 0.20µM FCA126, 0.20µM FCA090, 0.14µM Zn), 0.02 µL H_2_O, and 1.8 µL DNA extract. Multiplex 2 consisted of 3.5 µL 1 x concentrated Qiagen Master Mix, 0.7 µL of 0.5 x concentrated Qiagen Q solution, 0.85 µL of primers (0.20µM for F85, 0.09µM for F98, 0.11µM for FCA741, 0.43µM for FCA225, 0.10µM for FCA008, 0.29µM for Amel), 0.15 µL H_2_O, and 1.8 µL DNA extract. Multiplex 3 only differed in the amount of water (0.76 µL) and primers (0.24 µL; 0.20µM for F53, 0.14µM for FCA441) added. Microsatellite PCR amplifications were conducted using a DNA Engine Tetrad 2 Peltier Thermal Cycler (Bio-Rad Laboratories, Inc.) starting with an initial denaturation step of 15 min at 95 °C; followed by 13 cycles of 30 s at 94 °C for denaturation, 1.5 min at 60 °C with a decrease in annealing temperature of 0.8 °C in each cycle, and 1 min elongation at 72 °C; followed by 30 cycles of 30 s at 94 °C for denaturation, 1.5 min at 50 °C for annealing, and 1 min elongation at 72 °C; and 30 min at 60 °C for final elongation. A polymerase chain reaction negative was included in each group of PCR reactions to indicate the presence/absence of contamination. We visualized PCR products using an ABI PRISM^®^ 377 automated DNA sequencer (Applied Biosystems™), and genotypes were identified using the software GENEMAPPER, version 3.7 (Applied Biosystems™).

References

1. Wultsch C, Waits L, Kelly M. Noninvasive individual and species identification of jaguars (*Panthera onca*), pumas (*Puma concolor*) and ocelots (*Leopardus pardalis*) in Belize, Central America using cross-species microsatellites and fecal DNA. Mol Ecol Resour. 2014;14(6):1171-82.

2. Menotti-Raymond M, David VA, Lyons LA, Schaffer AA, Tomlin JF, Hutton MK, et al. A genetic linkage map of microsatellites in the domestic cat (*Felis catus*). Genomics. 1999;57(1):9-23.

3. Menotti-Raymond M, David V, Wachter L, Butler J, O'Brien S. An STIR forensic typing system for genetic individualization of domestic cat (*Felis catus*) samples. J Forensic Sci. 2005;50(5):1061-70.

4. Pilgrim KL, McKelvey KS, Riddle AE, Schwartz MK. Felid sex identification based on noninvasive genetic samples. Molecular Ecology Notes. 2005;5(1):60-1.
